# Mitochondria as multifaceted regulators of ferroptosis

**DOI:** 10.1093/lifemeta/loac035

**Published:** 2022-11-25

**Authors:** Jingyi Guo, Yunhao Zhou, Dingfei Liu, Mengfei Wang, Yi Wu, Daolin Tang, Xingguo Liu

**Affiliations:** CAS Key Laboratory of Regenerative Biology, Joint School of Life Sciences, Guangzhou Institutes of Biomedicine and Health, Chinese Academy of Sciences; Guangzhou Medical University, Guangzhou, Guangdong 510530, China; Guangdong Provincial Key Laboratory of Stem Cell and Regenerative Medicine, China-New Zealand Joint Laboratory on Biomedicine and Health, CUHK-GIBH Joint Research Laboratory on Stem Cells and Regenerative Medicine, Institute for Stem Cell and Regeneration, Guangzhou Institutes of Biomedicine and Health, Chinese Academy of Sciences, Guangzhou, Guangdong 510530, China; CAS Key Laboratory of Regenerative Biology, Joint School of Life Sciences, Guangzhou Institutes of Biomedicine and Health, Chinese Academy of Sciences; Guangzhou Medical University, Guangzhou, Guangdong 510530, China; Guangdong Provincial Key Laboratory of Stem Cell and Regenerative Medicine, China-New Zealand Joint Laboratory on Biomedicine and Health, CUHK-GIBH Joint Research Laboratory on Stem Cells and Regenerative Medicine, Institute for Stem Cell and Regeneration, Guangzhou Institutes of Biomedicine and Health, Chinese Academy of Sciences, Guangzhou, Guangdong 510530, China; University of Chinese Academy of Sciences, Beijing 100093, China; CAS Key Laboratory of Regenerative Biology, Joint School of Life Sciences, Guangzhou Institutes of Biomedicine and Health, Chinese Academy of Sciences; Guangzhou Medical University, Guangzhou, Guangdong 510530, China; Guangdong Provincial Key Laboratory of Stem Cell and Regenerative Medicine, China-New Zealand Joint Laboratory on Biomedicine and Health, CUHK-GIBH Joint Research Laboratory on Stem Cells and Regenerative Medicine, Institute for Stem Cell and Regeneration, Guangzhou Institutes of Biomedicine and Health, Chinese Academy of Sciences, Guangzhou, Guangdong 510530, China; University of Chinese Academy of Sciences, Beijing 100093, China; CAS Key Laboratory of Regenerative Biology, Joint School of Life Sciences, Guangzhou Institutes of Biomedicine and Health, Chinese Academy of Sciences; Guangzhou Medical University, Guangzhou, Guangdong 510530, China; Guangdong Provincial Key Laboratory of Stem Cell and Regenerative Medicine, China-New Zealand Joint Laboratory on Biomedicine and Health, CUHK-GIBH Joint Research Laboratory on Stem Cells and Regenerative Medicine, Institute for Stem Cell and Regeneration, Guangzhou Institutes of Biomedicine and Health, Chinese Academy of Sciences, Guangzhou, Guangdong 510530, China; University of Chinese Academy of Sciences, Beijing 100093, China; CAS Key Laboratory of Regenerative Biology, Joint School of Life Sciences, Guangzhou Institutes of Biomedicine and Health, Chinese Academy of Sciences; Guangzhou Medical University, Guangzhou, Guangdong 510530, China; Guangdong Provincial Key Laboratory of Stem Cell and Regenerative Medicine, China-New Zealand Joint Laboratory on Biomedicine and Health, CUHK-GIBH Joint Research Laboratory on Stem Cells and Regenerative Medicine, Institute for Stem Cell and Regeneration, Guangzhou Institutes of Biomedicine and Health, Chinese Academy of Sciences, Guangzhou, Guangdong 510530, China; Department of Surgery, UT Southwestern Medical Center, Dallas, TX, USA; CAS Key Laboratory of Regenerative Biology, Joint School of Life Sciences, Guangzhou Institutes of Biomedicine and Health, Chinese Academy of Sciences; Guangzhou Medical University, Guangzhou, Guangdong 510530, China; Guangdong Provincial Key Laboratory of Stem Cell and Regenerative Medicine, China-New Zealand Joint Laboratory on Biomedicine and Health, CUHK-GIBH Joint Research Laboratory on Stem Cells and Regenerative Medicine, Institute for Stem Cell and Regeneration, Guangzhou Institutes of Biomedicine and Health, Chinese Academy of Sciences, Guangzhou, Guangdong 510530, China; Centre for Regenerative Medicine and Health, Hong Kong Institute of Science & Innovation, Chinese Academy of Sciences, Hong Kong, China

**Keywords:** mitochondria, ferroptosis, lipid peroxidation, iron, ROS

## Abstract

Mitochondria are well known to be “energy factories” of the cell as they provide intracellular ATP via oxidative phosphorylation. Interestingly, they also function as a “cellular suicidal weapon store” by acting as a key mediator of various forms of regulated cell death, including apoptosis, pyroptosis, necroptosis, and ferroptosis. Ferroptosis, distinct from the other types of regulated cell death, is characterized by iron-dependent lipid peroxidation and subsequent plasma membrane rupture. Growing evidence suggests that an impaired ferroptotic response is implicated in various diseases and pathological conditions, and this impaired response is associated with dramatic changes in mitochondrial morphology and function. Mitochondria are the center of iron metabolism and energy production, leading to altered lipid peroxidation sensitivity. Although a growing number of studies have explored the inextricable link between mitochondria and ferroptosis, the role of this organelle in regulating ferroptosis remains unclear. Here, we review recent advances in our understanding of the role of mitochondria in ferroptosis and summarize the characteristics of this novel iron-based cellular suicide weapon and its arsenal. We also discuss the importance of ferroptosis in pathophysiology, including the need for further understanding of the relationship between mitochondria and ferroptosis to identify combinatorial targets that are essential for the development of successful drug discovery.

## Graphical Abstract

**Graphical Abstract:**
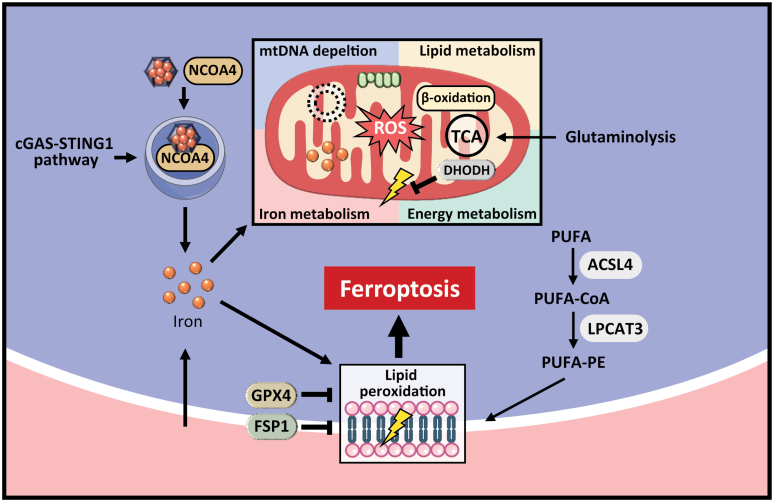

## Introduction

**Figure 1:**
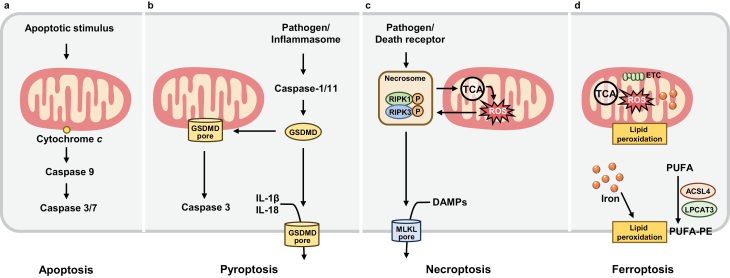
Mitochondria participate in cell death. Mitochondria participate in multiple forms of programmed cell death. (a) Mitochondria participate in the mitochondrial pathway of apoptosis. MOMP causes cytochrome *c* release, and subsequently causes caspase 9 and caspase 3/7 activation. (b) Pyroptosis is initiated by different inflammation-associated caspases, especially caspase-1. GSDMD is also localized to the mitochondria to induce MOMP, promoting the release of cytochrome *c* from mitochondria to the cytoplasm and further the activation of caspase-3. (c) Viral infection and TNF signaling activate RIPK1 and RIPK3, which form necrosomes. The necrosome then activates MLKL, which translocates to the plasma membrane to form pores, causing the release of DAMPs and cell death. RIPK3 also activates the PDHC, leading to TCA cycle enhancement and ROS generation. Mitochondrial ROS can promote RIPK1 autophosphorylation and necrosome formation to initiate necroptosis in a feedforward manner. (d) Ferroptosis is iron-dependent cell death triggered by lipid peroxidation accumulation that occurs in the plasma membrane. Abbreviations: PDHC, pyruvate dehydrogenase complex.

Mitochondria are semiautonomous double membrane-bound organelles that have a circular genome, referred to as mitochondrial DNA (mtDNA), and two separate outer membranes (OMMs) and inner membranes (IMMs) [1]. Human mtDNA contains genetic coding information for 13 proteins, which are core constituents of the electron transport chain (ETC) complexes I–IV, which are localized in IMMs. Together with the Krebs’ cycle in the matrix, the ETC creates an electrochemical gradient across the IMMs which powers the complex V, ATP synthase, to catalyze the synthesis of the largest pool of cellular ATP. This process involves the metabolism of carbon sources and is referred to as oxidative phosphorylation (OXPHOS) [2]. Mitochondria are crucial for cellular metabolism in eukaryotic organisms, and are involved in most forms of this cellular behavior, including cellular respiration, energy production, fatty acid oxidation, and iron metabolism. Mitochondria are critical for iron metabolism, as they are the major organelle involved in iron utilization and its catabolic and anabolic pathways. Mitochondria are highly dynamic as they take on a variety of morphologies controlled by the processes of fusion, fission, and mitophagy under a variety of physiological or pathological conditions. This plasticity is important for mitochondrial inheritance and function as it allows the cell to respond to ever-changing physiological conditions [3]. Mitochondria are also an important source of reactive oxygen species (ROS), which can lead to oxidative stress and even damage to the cell. In addition to their core metabolic functions, mitochondria have been implicated in a series of cellular processes, ranging from somatic cell reprogramming to cell death [4–7]. Although there are various forms of regulated cell death with different upstream signals and mechanisms [8], a role for the mitochondria is a common feature to all the forms (Fig. 1).

Mitochondria participate in the intrinsic pathway of apoptosis. Mitochondrial outer membrane permeabilization (MOMP) causes cytochrome *c* release, and subsequently causes caspase 9 and caspase 3/7 activation [9] (Fig. 1a). A widely used drug in epilepsy treatment, valproic acid, can induce mitochondria-dependent apoptosis, which causes liver failure in a large number of patients with Alpers-Huttenlocher syndrome [10], a disease due to mutations in *POLG*, which encodes for the alpha subunit of polymerase gamma. Pyroptosis is a proinflammatory-induced form of programmed cell death that is initiated by different inflammation-associated caspases, especially caspase-1, and it is marked by the rupture of the cell membrane due to perforations by Gasdermin D (GSDMD) and the release of proinflammatory mediators [11] (Fig. 1b). In this form of cell death, GSDMD is also localized to the mitochondria to induce MOMP, promoting the release of cytochrome *c* from mitochondria to the cytoplasm and further the activation of caspase-3 [12] (Fig. 1b). Necroptosis is a regulated caspase-independent form of cell death that is stimulated by viral infection and tumor necrosis factor (TNF) signaling [13]. In brief, under caspase inhibition, TNF signaling activates receptor interacting protein kinase 1 (RIPK1) and RIPK3, which form necrosomes. The necrosome then activates pseudokinase mixed-lineage kinase domain-like pseudokinase (MLKL), which translocates to the plasma membrane to form pores, causing the release of damage-associated molecular patterns (DAMPs) and cell death [14] (Fig. 1c). RIPK3 also activates the pyruvate dehydrogenase complex, leading to tricarboxylic acid (TCA) cycle enhancement and ROS generation. Mitochondrial ROS can promote RIPK1 autophosphorylation and necrosome formation to initiate necroptosis in a feedforward manner [9, 15] (Fig. 1c). Ferroptosis is an iron-dependent form of cell death that is triggered by lipid peroxidation accumulation, which occurs in the plasma membrane or in membranes of mitochondria, ER, lysosomes, and lipid droplets [16–18] (Fig. 1d). Mitochondria are major sites of ROS production and iron storage, and are thus susceptible to lipid peroxidation.

**Figure 2:**
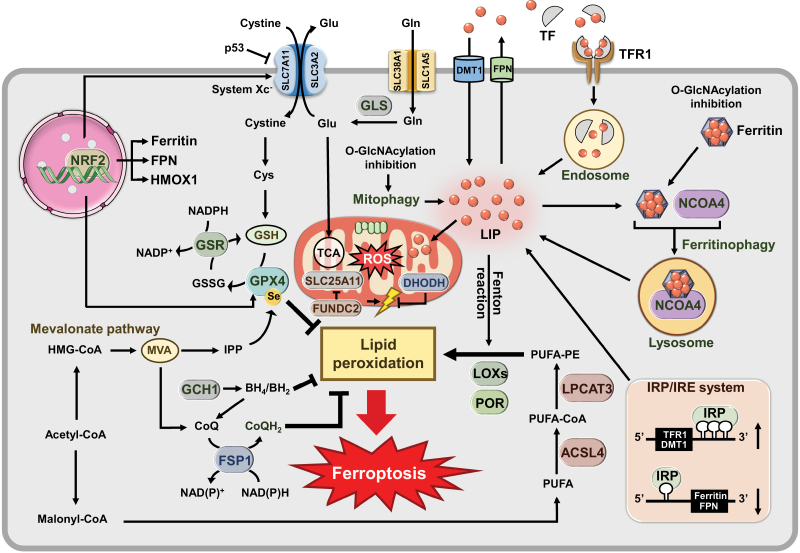
Main mechanisms of ferroptosis. Ferroptosis is triggered by peroxidation of PUFA-PE, induced by the Fenton reaction of iron, LOXs, and POR. ACSL4 and LPCAT3 are necessary for producing PUFA-PE. GPX4 requires GSH as a cofactor to inhibit lipid peroxidation, and GSR reduces GSSG to GSH. System Xc^−^ exchanges cystine and Glu, and cystine converts to Cys for GSH synthesis. Gln is converted to Glu through GLS and enters the mitochondria, which is required for ferroptosis triggered by Cys deprivation. FSP1 reduces CoQ to CoQH_2_, which inhibits lipid peroxidation. The MVA pathway participates in CoQ production and incorporation of Se into GPX4. DHODH, which reduces CoQ to CoQH_2_ in mitochondria, detoxifies lipid peroxides and inhibits ferroptosis. FUNDC2 regulates ferroptosis by interacting with SLC25A11 to regulate mitoGSH levels. GCH1, the rate-limiting enzyme for BH_4_/BH_2_, remodels lipids to suppress ferroptosis. BH_4_ can be eventually converted into 4-OH-benzoate, a precursor of CoQ, to elevate CoQ levels. Iron binds to TF to enter cells via TFR1 and localizes in endosomes, then iron is released into the LIP in the cytoplasm. Iron can also enter cells through DMT1 and can be exported from cells through FPN. Iron is utilized in the mitochondria for heme and Fe–S clusters synthesis. Excess iron is stored in ferritin. Iron is released from ferritin in lysosomes by NCOA4, which is known as ferritinophagy. De-*O*-GlcNAcylation of ferritin promotes ferritinophagy by increasing the interaction between ferritin and NCOA4, thereby accumulating labile iron. Cellular iron content can be regulated by the IRP/IRE system. NRF2 upregulates the expression of genes involved in GSH metabolism and iron metabolism. Abbreviations: Cys, cysteine; Glu, glutamate; Gln; glutamine; GSH, glutathione; GSSH, glutathione disulfide; GSR, glutathione disulfide reductase; HMG-CoA, 3-Hydroxy-3-Methyl-Glutaryl-CoA; IPP, isopentenyl diphosphate; Se, selenium; NRF2, nuclear factor E2-related factor 2.

Recently, insights into the mechanisms of ferroptosis have rapidly progressed [19–21] (Fig. 2). Glutamate and cystine are exchanged through an amino acid anti-transporter, system Xc^−^ [4]. Glutathione peroxidases (GPXs), especially GPX4, convert glutathione (GSH) into oxidized glutathione (GSSG) [22]. P53 can inhibit the activity of system Xc^−^ and thus reduces the absorption of cystine, leading to a decrease in GPX4 activity. As antioxidant capacity decreases, oxidative damage continues to increase and ultimately leads to ferroptosis [23–25] (Fig. 2). Ferroptosis suppressor protein 1 (FSP1) is characterized as a potent ferroptosis-resistance factor that reduces coenzyme Q_10_ (CoQ), a lipophilic radical-trapping antioxidant that eliminates lipid peroxides on plasma membrane. The FSP1–CoQ–NAD(P)H pathway is a parallel system to the canonical GSH-GPX4 pathway [26, 27] (Fig. 2).

The mevalonate (MVA) pathway can affect the synthesis of GPX4 by regulating the maturation of selenocysteine tRNA, thereby regulating the occurrence of ferroptosis [28, 29] (Fig. 2). Under conditions of GPX4 inactivation, dihydroorotate dehydrogenase (DHODH), which reduces CoQ to CoQH_2_ in mitochondria, detoxifies lipid peroxides and inhibits ferroptosis. This pathway acts parallel to mitochondrial GPX4 to inhibit ferroptosis, and is independent of GPX4 or FSP1 in the cytosol [30]. The OMM protein FUN14 domain containing 2 (FUNDC2) regulates ferroptosis by interacting with solute carrier family 25 member 11 (SLC25A11) to regulate mitochondrial GSH (mitoGSH) levels [31]. The FUNDC2-SLC25A11 axis serves as a newfound pathway in mitochondria to regulate ferroptosis (Fig. 2). Additionally, GTP cyclohydrolase-1 (GCH1), the rate-limiting enzyme that determines the levels of tetrahydrobiopterin/dihydrobiopterin (BH_4_/BH_2_), remodels lipids to suppress ferroptosis. BH_4_ can eventually be converted to 4-OH-benzoate, a precursor of CoQ, to elevate CoQ levels [32] (Fig. 2).

Two enzymes, acyl-CoA synthetase long-chain family member 4 (ACSL4) and lysophosphatidylcholine acyltransferase 3 (LPCAT3), are important drivers of ferroptosis. ACSL4 ligates long-chain polyunsaturated fatty acids (PUFAs), including arachidonic acid (AA) and adrenic acid (AdA), with coenzyme A (CoA). Then, the products are re-esterified into phospholipids (PLs) by LPCAT3 [33–35]. Peroxidation of PLs can be nonenzymatically catalyzed by labile free iron via the Fenton reaction or enzymatically catalyzed by lipoxygenases (LOXs) and cytochrome P450 oxidoreductase (POR) [36, 37] (Fig. 2). Fe^3+^ binds to transferrin (TF) on the cell membrane, which forms a complex with the membrane protein TF receptor 1 (TFR1) to allow for the endocytosis of the complex into endosomes [38] (Fig. 2). Unused or excreted iron in the cytoplasm is stored in ferritin, thus maintaining intracellular iron homeostasis. When iron is overloaded, the nuclear receptor coactivator 4 (NCOA4)-mediated autophagy process can selectively degrade ferritin via lysosomes, causing the level of intracellular free iron to be elevated [39] (Fig. 2). Protein *O*-GlcNAcylation, an important post-translational modification, has been shown to coordinate ferritinophagy with mitophagy to activate ferroptosis [40]. De-*O*-GlcNAcylation of ferritin promotes ferritinophagy by increasing the interaction between ferritin and NCOA4, thereby accumulating labile iron. Inhibition of *O*-GlcNAcylation enhances mitophagy, providing more labile iron, which make cells sensitive to ferroptosis [40] (Fig. 2). In addition, erastin induce the ferroptosis of liver cancer cells by inhibiting *O*-GlcNAcylated c-Jun, which has a positive correlation with GSH synthesis [41]. Overloaded labile iron then promotes Fenton chemistry reactions, which leads to the generation of toxic lipid alkoxy radicals and other reactive lipid breakdown products that drive ferroptosis [42].

Cellular iron content is regulated by the iron regulatory protein/iron-responsive element (IRP/IRE) system [43]. IRPs bind to IREs in the 5ʹ UTR of mRNAs to inhibit their translation, including that encode for ferritin and ferroportin (FPN), which are iron storage and iron export proteins, respectively. Alternatively, IRPs bind to IREs in the 3ʹ UTR of mRNAs, leading to stabilization of the mRNAs that encode for the iron uptake proteins TFR1 and divalent metal transporter 1 (DMT1) [44] (Fig. 2).

Induction of ferroptosis is associated with disruption of the balance of ROS production and antioxidant defense [45], as well as disorders of multiple signals about iron homeostasis, lipid synthesis, lipid peroxidation, or others from subcellular organelles [46]. Ferroptotic cells display morphological changes in their mitochondria, which is associated with increased mitochondrial membrane permeability [16, 47, 48]. Abnormal mitochondrial dynamics and dysfunctional mitochondria determine ferroptosis susceptibility [49]. Mitochondria also have unique mechanisms to defend against oxidative damage during ferroptosis. This review summarizes the recent advances in ferroptosis and emphasizes the role of mitochondria function and regulation in ferroptosis. Developing a better understanding of the relationship between ferroptosis and mitochondria will be conducive for disease therapies.

## Mitochondria and ferroptosis

**Figure 3:**
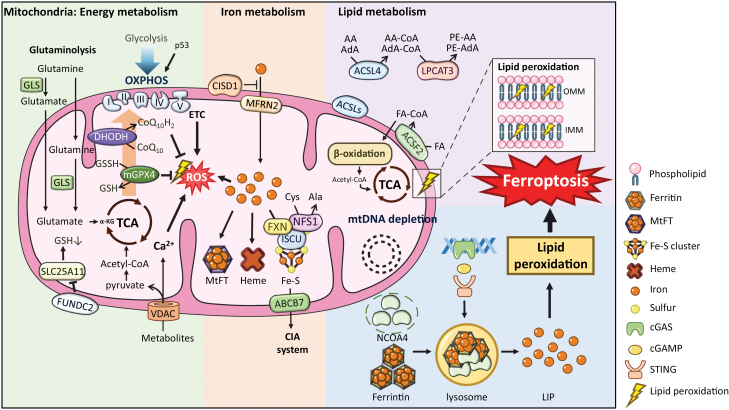
Regulation of mitochondria in ferroptosis. (1) The regulation of mitochondrial energy metabolism in ferroptosis. Cells with rerouting of metabolism from glycolysis to OXPHOS are sensitive to ferroptosis. FSP1 is an oxidoreductase reducing CoQ to CoQH_2_, which can inhibit lipid peroxidation. P53 directly regulates cellular metabolic versatility by favoring mitochondrial OXPHOS, leading to ROS-mediated ferroptosis. Glutamine is converted to glutamate by GLS, and glutamate is ultimately converted into α-KG in the TCA cycle. DHODH reduces CoQ to CoQH_2_ in mitochondria, which detoxifies lipid peroxides to protect cells from ferroptosis. It is a pathway parallel to mitoGPX4 in mitochondria to inhibit ferroptosis. The opening of VDAC, which mediates the entry of most metabolites into the mitochondria, increases mitochondrial metabolism and ROS formation. Ca^2+^ flows into the mitochondria through VDAC, contributing to ROS production. FUNDC2 regulates ferroptosis by interacting with SLC25A11 to regulate mitoGSH levels. The mitochondrial TCA cycle and ETC serve as the major source of lipid peroxide production. (2) The regulation of mitochondrial iron metabolism in ferroptosis. Mitochondria play a central role in cellular iron metabolism, and they are also the sole site of heme synthesis and the primary site of Fe–S cluster synthesis. Mitochondrial ROS interact with free iron and catalyze the Fenton reaction in mitochondria, which may generate more ROS. Excess iron can be stored in MtFT to protect mitochondria from free iron-induced damage. CISD1 can inhibit mitochondrial iron accumulation. Fe–S clusters are synthesized on a scaffold protein ISCU, and a cysteine desulfurase FNS1 supplies sulfur from Cys. NFS1 is important in maintaining the level of Fe–S clusters and protecting cells from ferroptosis. Fe–S clusters are exported to the cytosolic Fe–S assembly (CIA) system via ABCB7. (3) The regulation of mitochondrial lipid metabolism in ferroptosis. Oxidized phospholipids and cardiolipins mainly locate at the IMM in ferroptotic cells. Long-chain fatty acids are activated by ACSL4 to AA-CoA and AdA-CoA, which are esterified by LPCAT3 to phospholipids. ACSF2 and CS, which regulate mitochondrial fatty acid metabolism, both contribute to erastin-induced ferroptosis. (4) The regulation of mtDNA in ferroptosis. Hepatocytes with mtDNA depletion are more sensitive to iron overload-induced ferroptosis than parental cells with intact mtDNA. mtDNA depletion, ROS increase, GSH depletion, and NCOA4-mediated ferritin degradation in lysosomes, leading to a release of the LIP from ferritin, cause lipid peroxidation and eventual ferroptosis. mtDNA depletion and mtDNA stress also trigger autophagy-dependent ferroptosis through the activation of the cGAS-STING pathway. Intact mtDNA facilitates cellular defense against ferroptosis. Abbreviations: α-KG, α-ketoglutarate; Ala, alanine; FA, fatty acid.

Morphological changes of the mitochondria, including mitochondrial shrinkage, crista enlargement, and outer membrane rupture, have been observed in ferroptotic cells [16, 48]. The role of mitochondria in regulating cell ferroptosis is contentious according to a series of studies. Early studies reported that ferroptosis sensitivity was not affected by the loss of mtDNA or mitochondrial elimination [16, 50]. Recent studies, however, indicate that mitochondria play an important role in executing ferroptosis [51] (Fig. 3).

### Mitochondrial membrane and ferroptosis

The integrity of the mitochondrial membrane is critical for mitochondrial function. Ferroptosis is characterized by greater condensed densities of the mitochondrial membrane and a smaller volume compared to normal mitochondria, as well as diminished or even vanished cristae and ruptured OMMs. In RAS-selective lethal 3 (RSL3, a well-known inhibitor of GPX4)-induced ferroptosis, morphological alterations include elongation and fragmentation, with fragmented mitochondria mainly accumulating around the nucleus [51]. During ferroptosis, lipid oxidation occurs on the plasma membrane and the membranes of some organelles, including mitochondria [17, 18, 46]. It is unclear whether there is a prioritization of subcellular organelle membrane damage during ferroptosis.

#### Mitochondrial membrane lipids and ferroptosis

The main components of membrane lipids are PLs, sphingolipids, and sterols. PLs are amphiphilic molecules with a hydrophobic long hydrocarbon-based chain containing a saturated or unsaturated fatty acyl group at one end and a hydrophilic head composed of phosphatidylcholine (PC) and phosphatidylethanolamine (PE) [52]. During ferroptosis, lipid peroxidation preferentially occurs on PUFAs, which are straight-chain fatty acids with two or more double bonds and a carbon chain length of 18–22 carbon atoms. Peroxidation on PUFA-PLs results in alterations in lipid membrane structure and fluidity, and potentially forms hydrophilic pores that disrupt the plasma membrane barrier [53].

Oxidized PUFA-PCs and PUFA-PEs accumulate during ferroptosis in *Gpx4*^*−/−*^ mouse kidneys [48]. There are four species of oxidized arachidonic and adrenic PEs during RSL3-induced ferroptosis in mouse embryonic fibroblasts [17]. Mitochondria contain high levels of PE, and it is possible that lipid peroxidation may first occur on the OMMs and IMMs (Fig. 3). Oxidative modifications of cardiolipins (CLs), a mitochondria-specific phospholipids, were also observed in *Gpx4*^*−/−*^ mouse kidneys, and oxidized PLs and CLs were mainly localized to the IMM in ferroptotic cells [17, 18, 48]. Knockdown of *Smpd1* and *Taz*, which are responsible for CL synthesis, aggravated acetaminophen (APAP)-induced mitochondrial dysfunction and ferroptosis in hepatocytes [54]. However, oxidized CLs are considered to be pro-apoptotic signals that facilitate the release of cytochrome *c* from the mitochondria into the cytosol, executing intrinsic apoptosis [55]. The accumulation of oxidized CLs could not be inhibited by the lipophilic antioxidant ferrostatin-1, suggesting that oxidation of CLs is not sufficient to drive ferroptosis [17, 18]. Alternatively, cytochrome *c* can form distinct protein complexes to regulate apoptosis and ferroptosis, although both modes of cell death exhibit increased mitochondrial membrane permeability.

#### Mitochondrial membrane proteins and ferroptosis

Voltage-dependent anion channels (VDACs), also called mitochondrial porins, are located in the OMM and regulate mitochondrial metabolite transport. VDACs are considered as a convergence point of cell survival and death signals mediated by various ligands or proteins [56] (Fig. 3). VDAC opening leads to an increase in mitochondrial ROS, mitochondrial dysfunction, and subsequent oxidative stress-induced cell death [57]. Cells harboring oncogenic RAS with an abundance of VDACs are more sensitive to erastin, a small molecule capable of initiating ferroptosis, while knocking down VDAC2 or VDAC3 by RNAi can resist erastin-induced ferroptosis. Additionally, erastin directly binds to VDAC2 [58]. Notably, a study showed that inhibiting VDAC1 oligomerization attenuates hepatocyte ferroptosis by restoring ceramide and CLs content in APAP-induced liver injury [54]. As the VDAC family also plays a complex role in apoptosis, a further understanding of how they are modified and opened in ferroptosis is needed.

FUNDC2 was known to be an OMM-bound protein, but its biological functions were unclear. But recently, FUNDC2 was found to play an important role in regulating platelet activation through an AKT-GSK-3β-cGMP axis [59] and in mitochondria fragmentation by inhibiting mitofusin 1 (MFN1) [60]. Recently, it has been reported that FUNDC2 regulates ferroptosis by interacting with the mitochondrial glutathione transporter SLC25A11 to negatively regulate mitoGSH levels, and that it contributes to doxorubicin (DOX)-induced cardiomyopathy [31]. Under stimulation of ferroptosis, the interaction between FUNDC2 and SLC25A11 is enhanced, which reduces the stability and dimer formation of SLC25A11, resulting in a decrease in mitoGSH, leading to lipid peroxidation and ferroptosis (Fig. 3). FUNDC2 knockout protects mice from DOX-induced cardiomyopathy by preventing ferroptosis, and SLC25A11 knockdown reduces mitoGSH to prevent erastin-induced ferroptosis in FUNDC2-KO cells [31]. In short, the FUNDC2-SLC25A11 axis is a newfound pathway in mitochondria to regulate ferroptosis.

#### Mitochondrial lipid metabolism and ferroptosis

Two enzymes, ACSL4 and LPCAT3, are important drivers of ferroptosis. ACSL4 ligates long-chain PUFAs, including AA and AdA, with CoA. Then, the products are re-esterified into PLs by LPCAT3 [33–35] (Fig. 3). ACSL4 is not only a driver of ferroptosis but also a biomarker for predicting ferroptosis sensitivity [61]. Peroxidation of PLs is nonenzymatically catalyzed by labile free iron via the Fenton reaction, or enzymatically catalyzed by LOXs and POR [36, 37]. The ACSL family of proteins are localized to the ER and the OMM to catalyze the metabolism of fatty acids to acyl-CoA via β-oxidation (Fig. 3), with acyl-CoA being an intermediate product of lipid metabolism that participates in lipid biosynthesis and fatty acid degradation [62]. Dysregulation of ACSL inhibits or promotes apoptosis depending on the cell type [61]. It has been reported that ACSL4 is an essential part of ferroptosis execution. Breast cancer cell lines preferentially expressing ACSL4 are sensitive to ferroptosis [33]. ACSL5 is localized to the mitochondria, and it converts free long-chain fatty acids with 16–18 carbons, such as oleic acid, linoleic acid, and palmitic acid, to fatty acyl-coenzyme A. ACSL5 participates in pro-apoptotic sensing and acts as a tumor suppressor in cancers [63]. Presently, there is no evidence that ACSL5 plays a role in ferroptosis, and thus its role in that process requires further investigation. Acyl-CoA synthese family member 2 (ACSF2) catalyze the initial reaction in fatty acid metabolism by forming FA-CoA with FA and CoA [64] (Fig. 3). Study have found that ACSF2 and citrate synthase (CS), which regulate mitochondrial fatty acid metabolism, both contribute to erastin-induced ferroptosis [16]. In other contexts, ACSL3-mediated monounsaturated fatty acid (MUFA) production may lead to ferroptosis resistance [65], suggesting that different ACSL members have distinct functions in ferroptosis.

Fatty acid β-oxidation (FAO) is a process by which fatty acids are oxidized to sequentially remove two-carbon units from the acyl chain in mitochondria to metabolize the fatty acid to acetyl-CoA [66] (Fig. 3). 2,4-dienoyl-CoA reductase 1 (DECR1) is an auxiliary enzyme involved in PUFA β-oxidation. It has been reported that DECR1 knockdown inhibits PUFA β-oxidation and leads to accumulation of PUFAs in the LNCaP prostate cancer cell line. DECR1 knockdown-induced cell death is mediated by ferroptosis caused by accumulation of PUFAs in prostate cancer [67]. A similar conclusion was independently arrived at by a study showed that DECR1 knockout induces ER stress and sensitivity to ferroptosis in castration-resistant prostate cancer cells [68]. DECR1 participates in ferroptosis by controlling the balance between saturated and unsaturated fatty acids. The above studies may suggest that FAO protects cells from ferroptosis by reducing accumulation of PUFAs. FAO is a complicated process and understanding the molecular pathway by which it participates in ferroptosis is an interesting question for future investigations.

### Mitochondrial energy metabolism and ferroptosis

#### Mitochondrial OXPHOS and ferroptosis

The main function of mitochondria is to ensure energy production for cells by OXPHOS. Cells primarily rely on mitochondrial-based OXPHOS to generate energy for cellular processes, but most cancer cells rely on glycolysis, which is known as the “Warburg effect” [69]. Hexokinase II (HKII), platelet-type phosphofructokinase (PFKP), and pyruvate kinase M2 (PKM2) are key rate-limiting enzymes of glycolysis in cancer cells [70]. It has been reported that the levels of HKII, PFKP, and PKM2 are downregulated after treatment with RSL3 in glioma cells, indicating that RSL3 induces glycolytic dysfunction [71]. Various enzymes involved in mitochondrial respiration participate in the regulation of ferroptosis, such as aconitase, CS, cytochrome *c* oxidase 2 (sCO2), and fumarate hydratase (FH) [72]. NADPH-dependent ROS production is also important for the induction of ferroptosis, especially in the central nervous system [16]. Interestingly, mitochondria-deficient cells are sensitive to ferroptosis-inducing molecules, but are more tolerant to cysteine deprivation-induced ferroptosis [51].

Decreasing glycolytic flux, which is caused by increasing ATP synthesis and OXPHOS, was observed in erastin-induced ferroptosis [16]. The opening of VDACs, which mediates most of the metabolite entry into mitochondria, increases mitochondrial metabolism and thus ROS formation, subsequently leading to mitochondrial dysfunction [51]. As ferroptosis is related to system Xc^−^, the metabolites downstream of glutaminolysis will significantly decrease in the absence of glutamine, including αKG, fumarate, and malate [72]. Cells with a rerouting of cellular metabolism from glycolysis to OXPHOS are vulnerable to ferroptosis (Fig. 3). Under conditions of NRF2-mediated reactivation of OXPHOS, disrupting the cysteine transporter SLC7A11 will cause GSH depletion and subsequent cancer cell ferroptosis. These results suggest that the impermanentactivation of OXPHOS is related to mitochondrial oxidative damage in ferroptosis [73]. Furthermore, pyruvate oxidation-dependent fatty acid synthesis promotes ferroptosis, which can be inhibited by pyruvate dehydrogenase kinase 4 (PDK4) in pancreatic cancer cells [74]. Further understanding of the crosstalk between glucose metabolism, mitochondrial OXPHOS, and lipid metabolism may be the key to understanding the metabolic checkpoint of ferroptosis.

Mitochondria are major sites of cellular ROS production. ROS is produced in the ETC, in the form of superoxide, and is tightly regulated [75]. Cells treated with mitochondria-targeted antioxidants are rescued from the damage caused by lipid peroxidation. For example, the mitochondria-targeted antioxidant MitoTEMPO has been reported to rescue doxorubicin (DOX)-induced ferroptosis in cardiomyopathy, which suggests that mitochondria may be an important factor in DOX-induced ferroptosis in heart disease [76]. Another mitochondria-targeted antioxidant, nitroxide XJB-5-131, inhibits erastin- or RSL3-induced ferroptosis, suggesting that mitochondrial ROS plays a critical role in ferroptosis [77]. It has been reported that p53 is a positive regulator of ferroptosis by promoting ROS production [23]. It directly regulates cellular metabolic flexibility by favoring mitochondrial OXPHOS, leading to ROS-mediated ferroptosis. Nevertheless, under mild stress, p53 can protect cells by eliminating ROS. The mechanism of p53-mediated ROS production underlying the cellular response needs more investigation [78]. In addition, p53-mediated ferroptosis inhibition can be achieved by blocking dipeptidyl peptidase 4 activity to inhibit membrane lipid peroxidation [79]. Furthermore, some enzymes involved in mitochondrial respiration, including aconitase, CS, sCO2, and FH, are associated with the regulation of ferroptosis [72, 78, 80].

CoQ is an electron carrier in the mitochondrial ETC and a cellular endogenous lipophilic antioxidant. Ferroptosis inducing 56 (FIN56), a specific inducer of ferroptosis, activates squalene synthase to suppress CoQ, which enhances sensitivity to FIN56-induced ferroptosis [81]. Dihydroorotate dehydrogenase (DHODH) in the *de novo* pyrimidine synthesis pathway in mitochondria that generates uridine monophosphate, which yields pyrimidine for cell proliferation. DHODH converts dihydroorotate to orotate, which is the fourth step of the *de novo* pyrimidine synthesis pathway. DHODH links ETC via the CoQ pool and requires CoQ/ubiquinone as an electron acceptor [82]. DHODH can reduce CoQ to CoQH_2_ in mitochondria, which detoxifies lipid peroxides to protect cells from ferroptosis (Fig. 3). It is a pathway parallel to mitochondrial GPX4 to inhibit ferroptosis [83], and it is independent of GPX4 or FSP1 in the cytosol [30]. A further understanding of the functions of different mitochondrial electron carriers may be important for the development of new anticancer drugs.

#### Mitochondrial amino acid metabolism and ferroptosis

Glutaminolysis begins with glutamine being converted to glutamate by glutaminases (GLS), and glutamate is ultimately converted into TCA cycle metabolites in the mitochondria [84] (Fig. 3). Under normal conditions, glutamine and TF are essential for cell survival and growth. However, under conditions of amino acid starvation, glutamine and TF lead to ferroptosis [85].

Cysteine is a unique amino acid because of its redox-sensitive nature and as a source of GSH synthesis. Cellular cysteine is usually maintained at a low level due to its cytotoxic effects. However, cysteine is also required for GSH synthesis to maintain redox homeostasis, and it is converted to a series of bioactive molecules, such as taurine CoA, and Fe–S clusters [86]. The sulfur required for Fe–S cluster synthesis is provided by cysteine in the desulfurase complex NFS1-ISD11 [87] (Fig. 3). Additionally, mitochondria have been demonstrated to play an important role in cysteine deprivation-induced ferroptosis but not GPX4 inhibitor-induced ferroptosis. Inhibition of the mitochondrial ETC or TCA cycle mitigates cysteine deprivation-induced mitochondrial membrane potential hyperpolarization, lipid peroxidation, and ferroptosis [72]. Therefore, the above studies suggest that there are diverse roles for mitochondrial amino acid metabolism in regulating ferroptosis. The challenge is how to convert the pro-survival function of mitochondrial amino acid synthesis into a pro-cell death function.

#### Mitochondrial Ca^2+^ and ferroptosis

Increased intracellular Ca^2+^ and excessive ROS will cause an increase in mitochondrial Ca^2+^, mitochondrial fragmentation or potential mitochondrial membrane breakdown. Ruthenium red, an inhibitor of mitochondrial Ca^2+^ uptake, can block a high rate of ROS formation, suggesting that mitochondrial Ca^2+^ contributes to ROS production (Fig. 3). Recent studies have shown that compounds that inhibit oxidative glutamate toxicity by blocking mitochondrial ROS production or reducing Ca^2+^ influx can protect cells from ferroptosis induced by erastin, sulfasalazine, or another system Xc^−^ inhibitor [88]. DOX can trigger iron-mediated ROS production, mitochondrial dysfunction, and dysregulation of reticulum Ca^2+^ flux, which cause cardiotoxic effects [89]. In addition, it has been reported that the calcium chelator 1.2-bis(o-aminophenoxy)ethane-N,N,Nʹ,Nʹ-tetraacetic acid (BAPTA) effectively rescues erastin-induced ferroptosis in lung human mesencephalic cells [90]. These studies therefore suggest that mitochondrial Ca^2+^ plays an important role in inducing ferroptosis. Increased Ca^2+^ production may be the initial signal that triggers endosomal sorting complexes required for transport (ESCRT)-III-dependent membrane repair to inhibit ferroptosis [91, 92].

### Mitochondrial iron metabolism and ferroptosis

#### Mitochondrial iron and ferroptosis

Mitochondria contain ~20%–50% of the total pools of cellular iron and play a central role in cellular iron metabolism. They also provide compartmentalization, which is essential for strictly regulating cellular iron levels. The influx of cytoplasmic labile iron by mitoferrin1 and −2 is mainly utilized for the synthesis of heme and Fe–S clusters in mitochondria [93]. Excess iron can be stored in mitochondrial ferritin (MtFT), a mitochondrial-specific form of ferritin. Meanwhile, mitochondria are the main production sites of ROS by the ETC during ATP production. ROS can also interact with Fe–S clusters and catalyze the Fenton reaction in mitochondria, which may generate more ROS [44]. Consequently, a high level of iron and a high propensity for ROS generation lead mitochondria to become an ideal site for executing ferroptosis [94] (Fig. 3). 2,2-Bipyridyl (2,2-BP), a membrane-permeable iron chelator, can enter mitochondria and chelate mitochondrial iron, thus protecting HT-1080 cells from erastin-induced ferroptosis [16]. Another study also reported that 2,2-BP can inhibit glutaminolysis-induced ferroptosis [85]. In an ischemia-reperfusion injury mouse model, deferoxamine (DFO) and 2,2-BP both significantly decreased cellular iron levels. However, 2,2-BP, not DFO, can decrease mitochondrial labile iron and protect cells against oxidative damage in cardiac ischemia/reperfusion (I/R) injury [95]. These studies demonstrated that targeting mitochondria labile iron can protect cells from ferroptosis.

The NEET family is a group of proteins involved in a series of biological processes, including autophagy, apoptosis, and aging. In recent years, NEET proteins have been shown to play a role in the regulation of mitochondrial iron metabolism and ROS homeostasis. In humans, there are three genes known to encode NEET proteins. The OMM-localized protein mitoNEET [also referred to as CDGSH iron sulfur domain 1 (CISD1)] is essential for regulating mitochondrial function, iron metabolism, and ROS homeostasis [96] (Fig. 3). Loss of CISD1 results in mitochondrial iron accumulation and oxidative injury, ultimately inducing erastin-induced ferroptosis in cancer cells. In human hepatocellular carcinoma (HCC) cells, CISD1 negatively regulates ferroptosis by reducing mitochondrial iron accumulation to prevent mitochondrial damage [97]. In human epithelial breast cancer cells, the expression of CISD1 and CISD2 (also referred to as nutrient-deprivation autophagy factor-1) is elevated. Using shRNA to suppress the levels of CISD1 and CISD2 results in an increase in the accumulation of iron and ROS in mitochondria, resulting in a significant reduction in tumor growth [98]. In addition, silencing the CISD2 gene can overcome head and neck cancer cells’ resistance to sulfasalazine-induced ferroptosis by increasing the accumulation of mitochondrial iron and lipid peroxidation [99]. CISD3 was found to play a crucial role in cystine-deprivation-induced ferroptosis. CISD3 depletion results in a metabolic reprogramming toward glutaminolysis, which initiates ferroptosis [100]. These studies therefore suggest that the NEET proteins may serve as defense mechanisms against ferroptosis in mitochondria.

#### Iron–sulfur clusters and ferroptosis

Mitochondria are the primary generators of Fe–S clusters, which are essential cofactors for many proteins involved in cellular processes, including energy metabolism, DNA maintenance, lipid synthesis, and iron metabolism [101]. Fe–S clusters are first synthesized on a complex, which is composed of a scaffold protein ISCU, a cofactor protein ISD11 and a cysteine desulfurase NFS1 in mitochondria, requiring iron, cysteine, and electrons. Following *de novo* assembly, [2Fe–2S] clusters are released from the ISCU and transferred to glutaredoxin-related protein 5. Then, [2Fe–2S] clusters are directly inserted into [2Fe–2S] proteins or later [4Fe–4S] cluster. Additionally, Fe–S clusters are exported from mitochondria to the cytosol via the Fe–S cluster export machinery ABCB7 [102] (Fig. 3).

In the first stage of Fe–S cluster synthesis, sulfur along with iron are reassembled into Fe–S clusters, which are released from cysteine by NFS1. Sulfur insufficiency that cannot supply the NFS1 reaction will result in excessive free labile iron accumulation in mitochondria. It has been reported that NFS1 can protect lung tumor cells from ferroptosis. NFS1 is important in maintaining the level of Fe–S clusters. Insufficient Fe–S clusters lead to robust activation of the iron-starvation response, which, combined with GSH depletion, leads to triggering of ferroptosis [103]. ISCU plays a critical role in Fe–S cluster synthesis. Overexpression of ISCU significantly rescues dihydroartemisinin (DHA)-induced ferroptosis by rescuing mitochondrial function, regulating iron metabolism, and increasing cellular GSH levels [104]. Frataxin (FXN) is localized to the mitochondrial matrix and is an iron donor protein that transfers iron to the ISCU for the assembly of [2Fe–2S] clusters (Fig. 3). It is also a regulatory partner controlling sulfur production [105]. Friedreich’s ataxia (FRDA) is an autosomal recessive neurodegenerative disease that is characterized by progressive degeneration of the central and peripheral nervous systems and cardiomyopathy. FRDA is caused by the silencing of the *FXN* gene [106]. The decrease in FXN is associated with mitochondrial dysfunction, iron accumulation, and increased oxidative stress, which make patient cells more sensitive to erastin-induced ferroptosis [107]. Therefore, proteins involved in Fe–S cluster synthesis appear to represent a defense function against ferroptosis. Of note, abnormal Fe–S cluster synthesis is also associated with cuproptosis, a copper-triggered modality of mitochondrial cell death [108, 109]. One possibility is that different Fe–S proteins may selectively regulate ferroptosis and cuproptosis.

### mt DNA and ferroptosis

Human mtDNA is circular in form and is composed of 16,569 base pairs of circular DNA, and it is regulated by the nucleus. MtDNA encodes only two types of rRNA (12S and 16S), 22 types of tRNA and 13 types of polypeptides in mitochondrial ribosomes, which makes mitochondria semiautonomous [110]. The 13 polypeptides are essential components of the mitochondrial ETC and ATP synthase. Defects in mtDNA may impair mitochondrial respiratory function and cause a series of pathologies, aging, and death [111]. For instance, the accumulation of mtDNA mutations decreases female mouse fertility by impairing the NADH/NAD^+^ redox state in oocytes [112]. An “initial metabolic complementation” controlled by the interplay between mtDNA and mitochondrial fusion is a new mitochondrial function recovery strategy [113, 114].

It has been reported that a mtDNA-depleted (ρ0) cancer cell line is as sensitive to erastin- and RSL3-induced ferroptosis as mtDNA-wild-type cells [16]. However, there is also strong evidence that clearly shows the close relationship between mtDNA depletion and ferroptosis. Mitochondrial DNA depletion syndrome (MDS) is a group of autosomal recessive inherited disorders that are characterized by a severe reduction in mtDNA content in affected tissues and organs [115]. Patients with deoxyribonucleoside kinase (DGUOK) mutations usually die of severe liver failure before 2 years of age. There are no effective therapies except liver transplantation, leading to poor prognosis in almost all patients. A 3-D liver organoid disease model developed from iPSCs and derived from patients with MDS and with CRISPR/Cas9-mediated gene correction of the DGUOK mutation was established [116]. Hepatocytes with mtDNA depletion are more sensitive to iron overload-induced ferroptosis, which is an organelle interactive event between mitochondria and lysosomes. mtDNA depletion, ROS increase, GSH depletion, and NCOA4-mediated ferritin degradation in lysosomes that releases iron into the cytoplasm from ferritin cause lipid peroxidation and eventual ferroptosis [39] (Fig. 3). N-acetylcysteine, a precursor of GSH, significantly inhibited ferroptosis in hepatocytes from patients with MDS [116]. This is the first time that ferroptosis has been discussed in mitochondrial genetic diseases.

Pancreatic cancer is a serious gastrointestinal cancer with limited effective treatment and a very low 5-year overall survival rate [117]. Zalcitabine, an antiviral drug for treating human immunodeficiency virus infection, was reported to suppress human pancreatic cancer cell growth through inducing ferroptosis. Zalcitabine causes a reduction in mtDNA copy number, mitochondrial dysfunction, and increased total ROS. This mtDNA stress caused by zalcitabine triggers autophagy-dependent ferroptosis in human pancreatic cancer cells via the cGAS-STING pathway [118] (Fig. 3). STING1 is a transmembrane protein localized on the ER membrane and is closely related to ferroptosis [119, 120]. These studies suggest a complex relationship between mtDNA and ferroptosis in different cell types. As noted above, early studies described that a ρ0 cancer cell line is as sensitive to erastin- and RSL3-induced ferroptosis as mtDNA-wild-type cells [16], while another study clearly shows that hepatocytes with mtDNA depletion are more sensitive to iron overload-induced ferroptosis than parental cells with intact mtDNA [116]. Another study revealed that mtDNA reduction caused by Zalcitabine increases mtDNA stress and triggers autophagy-dependent ferroptosis in human pancreatic cancer cells. In some ρ0 cancer cell lines, lipid peroxidation accumulation on nonmitochondrial membranes could be sufficient to trigger ferroptosis. However, in other cell types, mtDNA depletion or its escape from the mitochondria actively contributes to ferroptosis. A deeper understanding of the regulation of mtDNA in ferroptosis needs further investigation in the future.

### Mitochondrial dynamics and ferroptosis

Mitochondria are highly dynamic organelles, which undergo mitochondrial fusion and fission. The fusion of the OMM and IMM is mediated by two classes of dynamin-like proteins: mitofusin 1 and 2 (MFN1/2) and optic atrophy 1 (OPA1) [121]. Loss of MFN2 is associated with mitochondrial iron overload, and OPA1 cleavage appears when cellular iron overload occurs [101]. Erastin induces STING1 to bind MFN1/2 to trigger mitochondrial fusion, leading to lipid peroxidation and subsequent ferroptosis in human pancreatic cancer cell lines [122]. The finding showed a new mitochondrial fusion-dependent ferroptosis mechanism. Mitochondrial fission is regulated by dynamin-related protein 1 (DRP1) from the cytosol, which binds to four OMM proteins: mitochondrial fission factor, mitochondrial fission protein 1 (FIS1), mitochondrial elongation factor 1 (MIEF1/MiD51), and MIEF2/MiD49 [123]. Loss of FIS1 shows a relationship between iron chelation and mitochondrial elongation, while cellular iron overload is associated with DRP1 dephosphorylation at Ser637. In a recent study, cotreatment with low concentrations of erastin and celastrol, a pentacyclic nortriterpen quinone that is believed to be anti-inflammatory and an inducer of autophagy [124], markedly induced cell death through the activation of the ROS–mitochondrial fission–mitophagy signaling pathway in non-small cell lung cancer (NSCLC) cells [125].

Another important aspect of mitochondrial dynamics is mitophagy. Mitophagy is selective autophagic degradation of mitochondria, and the well-known pathways are a PTEN induced kinase 1 (PINK1)-Parkin-mediated degradation pathway and a special mitochondrial selection strategy dependent on organellar topology under starvation [126]. Current studies show that both autophagy and mitophagy contributes to ferroptosis [127]. Both PINK1 knockdown-inhibited mitophagy and DRP1 knockdown-induced mitochondrial filamentation can inhibit mitochondrial complex I inhibitor BAY 87-2243-induced necroptosis and ferroptosis in BRAF^V600E^ melanoma cell lines [128]. Together, these studies suggest that mitochondrial dynamics plays roles in ferroptosis, but specific mechanisms to explain these effects require future investigation.

### Ferroptosis and mitochondrial diseases

**Figure 4:**
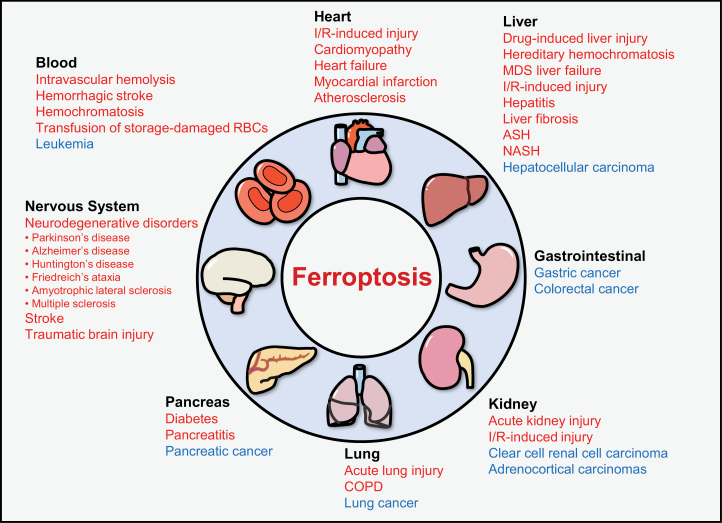
Ferroptosis and diseases. The relationship between ferroptosis and various diseases. Red color: Ferroptosis plays an important role in the occurrence and development of many diseases. Blue color: Ferroptosis has been confirmed as a potential treatment for some diseases. Abbreviations: I/R, ischemia/reperfusion; COPD, chronic obstructive pulmonary disease; RBCs, red blood cells.

The emergence of ferroptosis helps to explain the mechanisms of many drugs and diseases, as it is involved in a variety of biological processes. On the other hand, ferroptosis has been confirmed as a potential treatment in cancer and leukemia [129] (Fig. 4).

To date, only one article has explicitly reported that ferroptosis plays an important role in mitochondrial disease [116]. MDS is an autosomal recessive disorder with a broad genetic and clinical spectrum that is characterized by a significant decrease in mtDNA content in affected tissues and organs [115]. DGUOK is a 2-deoxyribonucleoside kinase that mediates the phosphorylation of deoxyguanosine and deoxyadenosinepurine in the corresponding nucleotides in mitochondria. DGUOK mutations are the main cause of hepatic encephalopathy during MDS, as they can cause an imbalance in the mitochondrial dNTP pool, which may lead to mtDNA synthesis breakdown and mtDNA depletion [130]. In addition, patients with DGUOK mutations show hepatic iron overload, which may progress to liver failure. Recent research revealed the connection between DGUOK mutations in MDS and iron overload-induced ferroptosis. DGUOK mutation-induced mtDNA depletion leads to mitochondrial dysfunction, reducing ATP production, and ROS enhancement, which cause GSH exhaustion. Meanwhile, a large quantity of the cellular labile iron pool (LIP) is released from ferritin, which is degraded in lysosomes in an NCOA4-dependent manner and subsequently leads to lipid peroxidation [39]. With the disruption of redox balance, the cells undergo ferroptosis.

Ferroptosis also plays an important role in the occurrence and development of many nonmitochondrial pathologies in different organs [131] (Fig. 4, color: red). In various heart diseases, fresh blood accompanied by an abundance of oxygen and iron triggers the Fenton reaction and the production of other peroxidation events, leading to the accumulation of ROS and ferroptosis during I/R-mediated injury [76, 132, 133], facilitating cardiomyopathy, myocardial infarction [134, 135], and atherosclerosis [136]. Unbalanced iron homeostasis can cause a variety of blood diseases. Ferroptosis is involved in the regulation of atherosclerosis, hemolysis, and some host immune impairments, such as hemochromatosis [137–139] and β-thalassemia [140]. In the immune system, GPX4 deficiency can induce T cell ferroptosis [141]. Ferroptosis plays a significant role in various brain diseases because iron accumulation is common to most of them [142]. Ferroptosis is a main cause of neuronal death after stroke [143–145], intracerebral hemorrhage [146], and traumatic brain injury [147]. In neurodegenerative diseases [148, 149], such as Alzheimer’s disease [150], iron accumulation in neuronal areas promotes the production of ROS. Ferroptosis plays an important role in many liver diseases, such as drug-induced liver injury [151, 152], hereditary hemochromatosis [153, 154], liver fibrosis [155], alcoholic steatohepatitis (ASH) [156], and non-alcoholic steatohepatitis (NASH) [157–159]. In addition, ferroptosis also contributes to many diseases of other organs, such as the kidney [48, 160–164], lung [103, 165–168], and pancreas [169–172].

Ferroptotic damage can promote tumor growth and it also has been confirmed as a potential treatment for some cancers, such as lung cancer, pancreatic cancer, and liver cancer [129] (Fig. 4, color: blue). In breast cancer, targeting the epithelial-mesenchymal transition (EMT)-MET process and cancer stem cells can trigger ferroptosis in cancer cells, thus inhibiting tumor growth and preventing invasion and metastasis [173–176], and lapatinib causes ferroptosis in breast cancer cells through iron accumulation by increasing the expression of TF and TFR and decreasing the expression of FPN [177]. In lung cancer, high levels of zinc can induce the depletion of GSSG and GSH, as well as the inactivation of system Xc^−^, inducing lipid peroxidation and ferroptosis, which has been proposed as a treatment for NSCLC [178]. In leukemia, RSL3 has been shown to induce ferroptosis in acute lymphoblastic leukemia cells by mediating LOXs, so it is expected to be a new therapeutic target for this condition [179].

## Conclusions and perspective

Ferroptosis is a unique form of cell death using an “iron weapon” to kill the cell, which involves many types of cellular metabolism, including those involving iron, lipids, ROS, and amino acids. Mitochondria not only act as “energy factories” that supply ATP for cellular activities, but also serve as “cellular suicidal weapon stores” that regulate cell death. To date, an increasing number of studies have uncovered a significant link between mitochondrial dysfunction and ferroptosis. In this review, we summarized the diverse mitochondrial processes, including mitochondrial lipid metabolism, energy metabolism, iron metabolism, mtDNA depletion, and mitochondrial dynamics, that have complex roles in regulating ferroptosis.

On one hand, mitochondria serve as a pro-ferroptosis factor. Mitochondria are a major source of ROS production, which may predispose cells to ferroptosis. Mitochondrial membrane lipids could be an ideal site of lipid peroxidation. The rerouting of cellular metabolism from glycolysis to OXPHOS renders cells susceptible to ferroptosis, and mitochondrial glutaminolysis contributes to ferroptosis triggered by amino acid deprivation. Mitochondria play a central role in iron metabolism, and high levels of iron make mitochondria the ideal place to execute ferroptosis. Early studies show that a ρ0 cancer cell line is as sensitive to ferroptosis as mtDNA-wild-type cells [16] and that cells depleted of mitochondria via mitophagy are still able to undergo ferroptosis [50]. Thus, whether mitochondria play an essential role in driving ferroptosis remains controversial.

On the other hand, mitochondria act as an anti-ferroptosis factor. FAO takes place mainly in mitochondria and β-oxidation inhibits lipid peroxidation by reducing accumulation of PUFAs. Furthermore, DHODH in mitochondria can protect cells from ferroptosis by detoxifying lipid peroxides under GPX4-inactivating conditions. Some proteins, such as NFS1 and FXN, involved in Fe–S cluster synthesis, appear to represent a defense function against ferroptosis. mtDNA depletion with subsequent mitochondrial dysfunction can increase the sensitivity to iron overload-induced ferroptosis in MDS. mtDNA depletion and mtDNA stress also trigger autophagy-dependent ferroptosis through the activation of the cGAS-STING1 pathway. Thus, intact and functional mitochondria facilitate cellular defense against ferroptosis. In conclusion, mitochondria-associated factors as pro-ferroptosis and anti-ferroptosis are summarized in Table 1.

**Table 1:** Mitochondria-associated molecules in ferroptosis

	Description	Model/Disease	References
Pro-ferroptosis factors			
ACSF2	ACSF2- and CS-dependent lipid synthesis pathways supply a lipid precursor required for ferroptosis	HT-1080 cells	[16]
CS			
DECR1	DECR1 knockdown inhibits PUFA β-oxidation and leads to accumulation of PUFAs	Prostate cancer cells	[67, 68]
PDK4	PDK4 dictates metabolic resistance to ferroptosis by suppressing pyruvate oxidation and fatty acid synthesis	Human pancreatic ductal carcinoma cells	[74]
VDACs	VDACs regulate mitochondrial metabolite transport; Erastin binds directly to VDAC2; Inhibiting VDAC1 oligomerization attenuates hepatocyte ferroptosis	Engineered human tumor cells APAP-induced liver injury	[54, 56, 58]
FUNDC2	FUNDC2 reduces the stability and dimer formation of SLC25A11, resulting in a decrease in mitoGSH and leading to lipid peroxidation and ferroptosis	DOX-induced cardiomyopathy	[31]
GLS	Glutamine is converted to glutamate by GLS. Under amino acid starvation conditions, glutamine leads to ferroptosis	Myocardial ischemic injury	[85]
Glutamate			
ROS	Mitochondria-targeted antioxidant MitoTEMPO rescues DOX-induced ferroptosis	DOX-induced cardiomyopathy	[75]
Ca^2+^	The calcium chelator BAPTA effectively rescues erastin-induced ferroptosis	Human lung mesencephalic cells	[90]
Iron	The mitochondria iron chelator 2,2-BP protects cells from ferroptosis	HT-1080 cells	[16, 84]
Anti-ferroptosis factors			
DHODH	DHODH reduces CoQ to CoQH_2_, which detoxifies lipid peroxides to protect cells from ferroptosis	Cancer cells	[82]
mGPX4	GPX4 in mitochondria protects cells from DOX-induced ferroptosis	DOX-induced cardiomyopathy	[83]
CISD1	CISD1 negatively regulates ferroptosis by reducing mitochondrial iron accumulation	HCC	[97]
ISCU	Overexpression of ISCU rescues DHA-induced ferroptosis	Leukemia cells	[104]
NFS1	NFS1 protects lung tumor cells from ferroptosis	Lung tumor cells	[103]
FXN	FXN is decreased in patient cells, leading to more sensitive to erastin-induced ferroptosis	FRDA	[107]
CLs	Knockdown of CLs-synthesizing enzymes aggravates APAP-induced mitochondrial dysfunction and ferroptosis in hepatocytes	APAP-induced Acute liver injury	[54]

The role of mitochondria in ferroptosis still faces many challenges, although mitochondrial dysfunction is a hallmark of cell death. For example, mitochondria are highly dynamic organelles, but the exact relationship between mitochondrial fission/fusion and ferroptosis is unclear. Moreover, mitochondria can contact other organelles, such as ER, lysosomes, plasma membrane, and peroxisomes [180]. It is unclear whether the membrane interactions and content exchanges between mitochondria and other organelles are involved in ferroptosis. Mitochondria can be transferred among cells by tunneling nanotubes (TNTs) [181]. Whether or not mitochondria transfer occurs and what is the regulatory effect of TNTs in ferroptosis is also still unclear. Aside from mitophagy, the functions of other mitochondrial quality control systems in ferroptosis remain poorly understood [182]. Overall, further studies are required to dissect the relevance of mitochondria with ferroptosis, and such efforts will likely be helpful in elucidating a further rationale for developing disease treatment strategies and mitochondria-targeted drugs.

## References

[1] Nunnari J , Suomalainen A. Mitochondria: in sickness and in health. Cell 2012;148:1145–59. 22424226 10.1016/j.cell.2012.02.035 PMC5381524

[2] Friedman JR , Nunnari J. Mitochondrial form and function. Nature 2014;505:335–43. 24429632 10.1038/nature12985 PMC4075653

[3] Westermann B. Mitochondrial fusion and fission in cell life and death. Nat Rev Mol Cell Biol 2010;11:872–84. 21102612 10.1038/nrm3013

[4] Ying Z , Chen K, Zheng L et al. Transient activation of mitoflashes modulates nanog at the early phase of somatic cell reprogramming. Cell Metab 2016;23:220–6. 26549484 10.1016/j.cmet.2015.10.002

[5] Ying Z , Xiang G, Zheng L et al. Short-term mitochondrial permeability transition pore opening modulates histone lysine methylation at the early phase of somatic cell reprogramming. Cell Metab 2018;28:935–45.e5. 30174306 10.1016/j.cmet.2018.08.001

[6] Xiang G , Yang L, Long Q et al. BNIP3L-dependent mitophagy accounts for mitochondrial clearance during 3 factors-induced somatic cell reprogramming. Autophagy 2017;13:1543–55. 28722510 10.1080/15548627.2017.1338545 PMC5612220

[7] Li L , Chen K, Wang T et al. Glis1 facilitates induction of pluripotency via an epigenome-metabolome-epigenome signalling cascade. Nat Metab 2020;2:882–92. 32839595 10.1038/s42255-020-0267-9

[8] Tang D , Kang R, Berghe TV et al. The molecular machinery of regulated cell death. Cell Res 2019;29:347–64. 30948788 10.1038/s41422-019-0164-5 PMC6796845

[9] Bock FJ , Tait SWG. Mitochondria as multifaceted regulators of cell death. Nat Rev Mol Cell Biol 2020;21:85–100. 31636403 10.1038/s41580-019-0173-8

[10] Li S , Guo J, Ying Z et al. Valproic acid-induced hepatotoxicity in Alpers syndrome is associated with mitochondrial permeability transition pore opening-dependent apoptotic sensitivity in an induced pluripotent stem cell model. Hepatology 2015;61:1730–9. 25605636 10.1002/hep.27712

[11] Shi J , Gao W, Shao F. Pyroptosis: gasdermin-mediated programmed necrotic cell death. Trends Biochem Sci 2017;42:245–54. 27932073 10.1016/j.tibs.2016.10.004

[12] Li Q , Shi N, Cai C et al. The role of mitochondria in pyroptosis. Front Cell Dev Biol 2020;8:630771. 33553170 10.3389/fcell.2020.630771 PMC7859326

[13] Yuan J , Amin P, Ofengeim D. Necroptosis and RIPK1-mediated neuroinflammation in CNS diseases. Nat Rev Neurosci 2019;20:19–33. 30467385 10.1038/s41583-018-0093-1 PMC6342007

[14] Gong Y , Fan Z, Luo G et al. The role of necroptosis in cancer biology and therapy. Mol Cancer 2019;18:100. 31122251 10.1186/s12943-019-1029-8 PMC6532150

[15] Xue C , Gu X, Li G et al. Mitochondrial mechanisms of necroptosis in liver diseases. Int J Mol Sci 2020;22:66. 33374660 10.3390/ijms22010066 PMC7793526

[16] Dixon SJ , Lemberg KM, Lamprecht MR et al. Ferroptosis: an iron-dependent form of nonapoptotic cell death. Cell 2012;149:1060–72. 22632970 10.1016/j.cell.2012.03.042 PMC3367386

[17] Kagan VE , Mao G, Qu F et al. Oxidized arachidonic and adrenic PEs navigate cells to ferroptosis. Nat Chem Biol 2017;13:81–90. 27842066 10.1038/nchembio.2238 PMC5506843

[18] Gaschler MM , Andia AA, Liu H et al. FINO2 initiates ferroptosis through GPX4 inactivation and iron oxidation. Nat Chem Biol 2018;14:507–15. 29610484 10.1038/s41589-018-0031-6 PMC5899674

[19] Chen X , Li J, Kang R et al. Ferroptosis: machinery and regulation. Autophagy 2021;17:2054–81. 32804006 10.1080/15548627.2020.1810918 PMC8496712

[20] Stockwell BR , Friedmann Angeli JP, Bayir H et al. Ferroptosis: a regulated cell death nexus linking metabolism, redox biology, and disease. Cell 2017;171:273–85. 28985560 10.1016/j.cell.2017.09.021 PMC5685180

[21] Stockwell BR. Ferroptosis turns 10: emerging mechanisms, physiological functions, and therapeutic applications. Cell 2022;185:2401–21. 35803244 10.1016/j.cell.2022.06.003 PMC9273022

[22] Brigelius-Flohe R , Maiorino M. Glutathione peroxidases. Biochim Biophys Acta 2013;1830:3289–303. 23201771 10.1016/j.bbagen.2012.11.020

[23] Jiang L , Kon N, Li T et al. Ferroptosis as a p53-mediated activity during tumour suppression. Nature 2015;520:57–62. 25799988 10.1038/nature14344 PMC4455927

[24] Jiang L , Hickman JH, Wang SJ et al. Dynamic roles of p53-mediated metabolic activities in ROS-induced stress responses. Cell Cycle (Georgetown, Tex) 2015;14:2881–5. 26218928 10.1080/15384101.2015.1068479 PMC4825584

[25] Yang WS , SriRamaratnam R, Welsch ME et al. Regulation of ferroptotic cancer cell death by GPX4. Cell 2014;156:317–31. 24439385 10.1016/j.cell.2013.12.010 PMC4076414

[26] Doll S , Freitas FP, Shah R et al. FSP1 is a glutathione-independent ferroptosis suppressor. Nature 2019;575:693–8. 31634899 10.1038/s41586-019-1707-0

[27] Bersuker K , Hendricks JM, Li Z et al. The CoQ oxidoreductase FSP1 acts parallel to GPX4 to inhibit ferroptosis. Nature 2019;575:688–92. 31634900 10.1038/s41586-019-1705-2 PMC6883167

[28] Warner GJ , Berry MJ, Moustafa ME et al. Inhibition of selenoprotein synthesis by selenocysteine tRNA[Ser]Sec lacking isopentenyladenosine. J Biol Chem 2000;275:28110–9. 10821829 10.1074/jbc.M001280200

[29] Venkatesh D , O’Brien NA, Zandkarimi F et al. MDM2 and MDMX promote ferroptosis by PPARα-mediated lipid remodeling. Genes Dev 2020;34:526–43. 32079652 10.1101/gad.334219.119 PMC7111265

[30] Mao C , Liu X, Zhang Y et al. DHODH-mediated ferroptosis defence is a targetable vulnerability in cancer. Nature 2021;593:586–90. 33981038 10.1038/s41586-021-03539-7 PMC8895686

[31] Ta N , Qu C, Wu H et al. Mitochondrial outer membrane protein FUNDC2 promotes ferroptosis and contributes to doxorubicin-induced cardiomyopathy. Proc Natl Acad Sci USA 2022;119:e2117396119. 36037337 10.1073/pnas.2117396119 PMC9457330

[32] Kraft VAN , Bezjian CT, Pfeiffer S et al. GTP cyclohydrolase 1/tetrahydrobiopterin counteract ferroptosis through lipid remodeling. ACS Cent Sci 2020;6:41–53. 31989025 10.1021/acscentsci.9b01063 PMC6978838

[33] Doll S , Proneth B, Tyurina YY et al. ACSL4 dictates ferroptosis sensitivity by shaping cellular lipid composition. Nat Chem Biol 2017;13:91–8. 27842070 10.1038/nchembio.2239 PMC5610546

[34] Zou Y , Palte MJ, Deik AA et al. A GPX4-dependent cancer cell state underlies the clear-cell morphology and confers sensitivity to ferroptosis. Nat Commun 2019;10:1617. 30962421 10.1038/s41467-019-09277-9 PMC6453886

[35] Dixon SJ , Winter GE, Musavi LS et al. Human haploid cell genetics reveals roles for lipid metabolism genes in nonapoptotic cell death. ACS Chem Biol 2015;10:1604–9. 25965523 10.1021/acschembio.5b00245 PMC4509420

[36] Jiang X , Stockwell BR, Conrad M. Ferroptosis: mechanisms, biology and role in disease. Nat Rev Mol Cell Biol 2021;22:266–82. 33495651 10.1038/s41580-020-00324-8 PMC8142022

[37] Wu H , Wang F, Ta N et al. The multifaceted regulation of mitochondria in ferroptosis. Life (Basel, Switzerland) 2021;11:222. 33801920 10.3390/life11030222 PMC8001967

[38] Frazer DM , Anderson GJ. The regulation of iron transport. BioFactors (Oxford, England) 2014;40:206–14. 24132807 10.1002/biof.1148

[39] Hou W , Xie Y, Song X et al. Autophagy promotes ferroptosis by degradation of ferritin. Autophagy 2016;12:1425–8. 27245739 10.1080/15548627.2016.1187366 PMC4968231

[40] Yu F , Zhang Q, Liu H et al. Dynamic O-GlcNAcylation coordinates ferritinophagy and mitophagy to activate ferroptosis. Cell Discov 2022;8:40. 35504898 10.1038/s41421-022-00390-6 PMC9065108

[41] Chen Y , Zhu G, Liu Y et al. O-GlcNAcylated c-Jun antagonizes ferroptosis via inhibiting GSH synthesis in liver cancer. Cell Signal 2019;63:109384. 31394193 10.1016/j.cellsig.2019.109384

[42] Hirschhorn T , Stockwell BR. The development of the concept of ferroptosis. Free Radic Biol Med 2019;133:130–43. 30268886 10.1016/j.freeradbiomed.2018.09.043 PMC6368883

[43] Muckenthaler MU , Rivella S, Hentze MW et al. A red carpet for iron metabolism. Cell 2017;168:344–61. 28129536 10.1016/j.cell.2016.12.034 PMC5706455

[44] Chen X , Yu C, Kang R et al. Iron metabolism in ferroptosis. Front Cell Dev Biol 2020;8:590226. 33117818 10.3389/fcell.2020.590226 PMC7575751

[45] Kuang F , Liu J, Tang D et al. Oxidative damage and antioxidant defense in ferroptosis. Front Cell Dev Biol 2020;8:586578. 33043019 10.3389/fcell.2020.586578 PMC7527737

[46] Chen X , Kang R, Kroemer G et al. Organelle-specific regulation of ferroptosis. Cell Death Differ 2021;28:2843–56. 34465893 10.1038/s41418-021-00859-z PMC8481335

[47] Xie Y , Hou W, Song X et al. Ferroptosis: process and function. Cell Death Differ 2016;23:369–79. 26794443 10.1038/cdd.2015.158 PMC5072448

[48] Friedmann Angeli JP , Schneider M, Proneth B et al. Inactivation of the ferroptosis regulator Gpx4 triggers acute renal failure in mice. Nat Cell Biol 2014;16:1180–91. 25402683 10.1038/ncb3064 PMC4894846

[49] Gan B. Mitochondrial regulation of ferroptosis. J Cell Biol 2021;220:e202105043. 34328510 10.1083/jcb.202105043 PMC8329737

[50] Gaschler MM , Hu F, Feng H et al. Determination of the subcellular localization and mechanism of action of ferrostatins in suppressing ferroptosis. ACS Chem Biol 2018;13:1013–20. 29512999 10.1021/acschembio.8b00199 PMC5960802

[51] Wang H , Liu C, Zhao Y et al. Mitochondria regulation in ferroptosis. Eur J Cell Biol 2020;99:151058. 31810634 10.1016/j.ejcb.2019.151058

[52] van Meer G , Voelker DR, Feigenson GW. Membrane lipids: where they are and how they behave. Nat Rev Mol Cell Biol 2008;9:112–24. 18216768 10.1038/nrm2330 PMC2642958

[53] Boonnoy P , Karttunen M, Wong-Ekkabut J. Alpha-tocopherol inhibits pore formation in oxidized bilayers. Phys Chem Chem Phys 2017;19:5699–704. 28138670 10.1039/c6cp08051k

[54] Niu B , Lei X, Xu Q et al. Protecting mitochondria via inhibiting VDAC1 oligomerization alleviates ferroptosis in acetaminophen-induced acute liver injury. Cell Biol Toxicol 2022;38:505–30. 34401974 10.1007/s10565-021-09624-x

[55] Tyurina YY , Shrivastava I, Tyurin VA et al. “Only a life lived for others is worth living”: redox signaling by oxygenated phospholipids in cell fate decisions. Antioxid Redox Signal 2018;29:1333–58. 28835115 10.1089/ars.2017.7124 PMC6157439

[56] Shoshan-Barmatz V , De Pinto V, Zweckstetter M et al. VDAC, a multi-functional mitochondrial protein regulating cell life and death. Mol Aspects Med 2010;31:227–85. 20346371 10.1016/j.mam.2010.03.002

[57] DeHart DN , Fang D, Heslop K et al. Opening of voltage dependent anion channels promotes reactive oxygen species generation, mitochondrial dysfunction and cell death in cancer cells. Biochem Pharmacol 2018;148:155–62. 29289511 10.1016/j.bcp.2017.12.022 PMC5909406

[58] Yagoda N , von Rechenberg M, Zaganjor E et al. RAS-RAF-MEK-dependent oxidative cell death involving voltage-dependent anion channels. Nature 2007;447:864–8. 17568748 10.1038/nature05859 PMC3047570

[59] Ma Q , Zhang W, Zhu C et al. FUNDC2 regulates platelet activation through AKT/GSK-3beta/cGMP axis. Cardiovasc Res 2019;115:1672–9. 30576423 10.1093/cvr/cvy311

[60] Li S , Han S, Zhang Q et al. FUNDC2 promotes liver tumorigenesis by inhibiting MFN1-mediated mitochondrial fusion. Nat Commun 2022;13:3486. 35710796 10.1038/s41467-022-31187-6 PMC9203792

[61] Yuan H , Li X, Zhang X et al. Identification of ACSL4 as a biomarker and contributor of ferroptosis. Biochem Biophys Res Commun 2016;478:1338–43. 27565726 10.1016/j.bbrc.2016.08.124

[62] Yan S , Yang XF, Liu HL et al. Long-chain acyl-CoA synthetase in fatty acid metabolism involved in liver and other diseases: an update. World J Gastroenterol 2015;21:3492–8. 25834313 10.3748/wjg.v21.i12.3492 PMC4375570

[63] Quan J , Bode AM, Luo X. ACSL family: the regulatory mechanisms and therapeutic implications in cancer. Eur J Pharmacol 2021;909:174397. 34332918 10.1016/j.ejphar.2021.174397

[64] Wang M , Su Y, Hou C et al. Targeted lipidomics analysis of lysine 179 acetylation of ACSF2 in rat hepatic stellate cells. Prostaglandins Other Lipid Mediat 2022;163:106671. 36028068 10.1016/j.prostaglandins.2022.106671

[65] Magtanong L , Ko PJ, To M et al. Exogenous monounsaturated fatty acids promote a ferroptosis-resistant cell state. Cell Chem Biol 2019;26:420–32.e9. 30686757 10.1016/j.chembiol.2018.11.016 PMC6430697

[66] Eaton S. Control of mitochondrial beta-oxidation flux. Prog Lipid Res 2002;41:197–239. 11814524 10.1016/s0163-7827(01)00024-8

[67] Nassar ZD , Mah CY, Dehairs J et al. Human DECR1 is an androgen-repressed survival factor that regulates PUFA oxidation to protect prostate tumor cells from ferroptosis. Elife 2020;9:e54166. 32686647 10.7554/eLife.54166 PMC7386908

[68] Blomme A , Ford CA, Mui E et al. 2,4-dienoyl-CoA reductase regulates lipid homeostasis in treatment-resistant prostate cancer. Nat Commun 2020;11:2508. 32427840 10.1038/s41467-020-16126-7 PMC7237503

[69] Vander Heiden MG , Cantley LC, Thompson CB. Understanding the Warburg effect: the metabolic requirements of cell proliferation. Science 2009;324:1029–33. 19460998 10.1126/science.1160809 PMC2849637

[70] Li XB , Gu JD, Zhou QH. Review of aerobic glycolysis and its key enzymes - new targets for lung cancer therapy. Thorac Cancer 2015;6:17–24. 26273330 10.1111/1759-7714.12148 PMC4448463

[71] Wang X , Lu S, He C et al. RSL3 induced autophagic death in glioma cells via causing glycolysis dysfunction. Biochem Biophys Res Commun 2019;518:590–7. 31445705 10.1016/j.bbrc.2019.08.096

[72] Gao M , Yi J, Zhu J et al. Role of mitochondria in ferroptosis. Mol Cell 2019;73:354–63.e3. 30581146 10.1016/j.molcel.2018.10.042 PMC6338496

[73] Zdralevic M , Vucetic M, Daher B et al. Disrupting the ‘Warburg effect’ re-routes cancer cells to OXPHOS offering a vulnerability point via ‘ferroptosis’-induced cell death. Adv Biol Regul 2018;68:55–63. 29306548 10.1016/j.jbior.2017.12.002

[74] Song X , Liu J, Kuang F et al. PDK4 dictates metabolic resistance to ferroptosis by suppressing pyruvate oxidation and fatty acid synthesis. Cell Rep 2021;34:108767. 33626342 10.1016/j.celrep.2021.108767

[75] Dan Dunn J , Alvarez LA, Zhang X et al. Reactive oxygen species and mitochondria: a nexus of cellular homeostasis. Redox Biol 2015;6:472–85. 26432659 10.1016/j.redox.2015.09.005 PMC4596921

[76] Fang X , Wang H, Han D et al. Ferroptosis as a target for protection against cardiomyopathy. Proc Natl Acad Sci USA 2019;116:2672–80. 30692261 10.1073/pnas.1821022116 PMC6377499

[77] Krainz T , Gaschler MM, Lim C et al. A mitochondrial-targeted nitroxide is a potent inhibitor of ferroptosis. ACS Cent Sci 2016;2:653–9. 27725964 10.1021/acscentsci.6b00199 PMC5043442

[78] Zhang W , Gai C, Ding D et al. Targeted p53 on small-molecules-induced ferroptosis in cancers. Front Oncol 2018;8:507. 30450337 10.3389/fonc.2018.00507 PMC6224449

[79] Xie Y , Zhu S, Song X et al. The tumor suppressor p53 limits ferroptosis by blocking DPP4 activity. Cell Rep 2017;20:1692–704. 28813679 10.1016/j.celrep.2017.07.055

[80] Venkatesh S , Li M, Saito T et al. Mitochondrial LonP1 protects cardiomyocytes from ischemia/reperfusion injury in vivo. J Mol Cell Cardiol 2019;128:38–50. 30625302 10.1016/j.yjmcc.2018.12.017

[81] Shimada K , Skouta R, Kaplan A et al. Global survey of cell death mechanisms reveals metabolic regulation of ferroptosis. Nat Chem Biol 2016;12:497–503. 27159577 10.1038/nchembio.2079 PMC4920070

[82] Boukalova S , Hubackova S, Milosevic M et al. Dihydroorotate dehydrogenase in oxidative phosphorylation and cancer. Biochim Biophys Acta Mol Basis Dis 2020;1866:165759. 32151633 10.1016/j.bbadis.2020.165759

[83] Tadokoro T , Ikeda M, Ide T et al. Mitochondria-dependent ferroptosis plays a pivotal role in doxorubicin cardiotoxicity. JCI Insight 2020;5:e132747. 32376803 10.1172/jci.insight.132747 PMC7253028

[84] Yang L , Venneti S, Nagrath D. Glutaminolysis: a hallmark of cancer metabolism. Annu Rev Biomed Eng 2017;19:163–94. 28301735 10.1146/annurev-bioeng-071516-044546

[85] Gao M , Monian P, Quadri N et al. Glutaminolysis and transferrin regulate ferroptosis. Mol Cell 2015;59:298–308. 26166707 10.1016/j.molcel.2015.06.011 PMC4506736

[86] Lill R , Dutkiewicz R, Freibert SA et al. The role of mitochondria and the CIA machinery in the maturation of cytosolic and nuclear iron-sulfur proteins. Eur J Cell Biol 2015;94:280–91. 26099175 10.1016/j.ejcb.2015.05.002

[87] Stehling O , Wilbrecht C, Lill R. Mitochondrial iron-sulfur protein biogenesis and human disease. Biochimie 2014;100:61–77. 24462711 10.1016/j.biochi.2014.01.010

[88] Maher P , van Leyen K, Dey PN et al. The role of Ca^2+^ in cell death caused by oxidative glutamate toxicity and ferroptosis. Cell Calcium 2018;70:47–55. 28545724 10.1016/j.ceca.2017.05.007 PMC5682235

[89] Thorn CF , Oshiro C, Marsh S et al. Doxorubicin pathways: pharmacodynamics and adverse effects. Pharmacogenet Genomics 2011;21:440–6. 21048526 10.1097/FPC.0b013e32833ffb56 PMC3116111

[90] Do Van B , Gouel F, Jonneaux A et al. Ferroptosis, a newly characterized form of cell death in Parkinson’s disease that is regulated by PKC. Neurobiol Dis 2016;94:169–78. 27189756 10.1016/j.nbd.2016.05.011

[91] Dai E , Meng L, Kang R et al. ESCRT-III-dependent membrane repair blocks ferroptosis. Biochem Biophys Res Commun 2020;522:415–21. 31761326 10.1016/j.bbrc.2019.11.110 PMC6957708

[92] Pedrera L , Espiritu RA, Ros U et al. Ferroptotic pores induce Ca^2+^ fluxes and ESCRT-III activation to modulate cell death kinetics. Cell Death Differ 2021;28:1644–57. 33335287 10.1038/s41418-020-00691-x PMC8167089

[93] Xu T , Ding W, Ji X et al. Molecular mechanisms of ferroptosis and its role in cancer therapy. J Cell Mol Med 2019;23:4900–12. 31232522 10.1111/jcmm.14511 PMC6653007

[94] Thomas C , Mackey MM, Diaz AA et al. Hydroxyl radical is produced via the Fenton reaction in submitochondrial particles under oxidative stress: implications for diseases associated with iron accumulation. Redox Rep 2009;14:102–8. 19490751 10.1179/135100009X392566

[95] Chang HC , Wu R, Shang M et al. Reduction in mitochondrial iron alleviates cardiac damage during injury. EMBO Mol Med 2016;8:247–67. 26896449 10.15252/emmm.201505748 PMC4772952

[96] Geldenhuys WJ , Leeper TC, Carroll RT. mitoNEET as a novel drug target for mitochondrial dysfunction. Drug Discov Today 2014;19:1601–6. 24814435 10.1016/j.drudis.2014.05.001

[97] Yuan H , Li X, Zhang X et al. CISD1 inhibits ferroptosis by protection against mitochondrial lipid peroxidation. Biochem Biophys Res Commun 2016;478:838–44. 27510639 10.1016/j.bbrc.2016.08.034

[98] Sohn YS , Tamir S, Song L et al. NAF-1 and mitoNEET are central to human breast cancer proliferation by maintaining mitochondrial homeostasis and promoting tumor growth. Proc Natl Acad Sci USA 2013;110:14676–81. 23959881 10.1073/pnas.1313198110 PMC3767537

[99] Kim EH , Shin D, Lee J et al. CISD2 inhibition overcomes resistance to sulfasalazine-induced ferroptotic cell death in head and neck cancer. Cancer Lett 2018;432:180–90. 29928961 10.1016/j.canlet.2018.06.018

[100] Li Y , Wang X, Huang Z et al. CISD3 inhibition drives cystine-deprivation induced ferroptosis. Cell Death Dis 2021;12:839. 34497268 10.1038/s41419-021-04128-2 PMC8426496

[101] Ward DM , Cloonan SM. Mitochondrial iron in human health and disease. Annu Rev Physiol 2019;81:453–82. 30485761 10.1146/annurev-physiol-020518-114742 PMC6641538

[102] Wachnowsky C , Fidai I, Cowan JA. Iron-sulfur cluster biosynthesis and trafficking - impact on human disease conditions. Metallomics 2018;10:9–29. 29019354 10.1039/c7mt00180k PMC5783746

[103] Alvarez SW , Sviderskiy VO, Terzi EM et al. NFS1 undergoes positive selection in lung tumours and protects cells from ferroptosis. Nature 2017;551:639–43. 29168506 10.1038/nature24637 PMC5808442

[104] Du J , Wang T, Li Y et al. DHA inhibits proliferation and induces ferroptosis of leukemia cells through autophagy dependent degradation of ferritin. Free Radic Biol Med 2019;131:356–69. 30557609 10.1016/j.freeradbiomed.2018.12.011

[105] Colin F , Martelli A, Clemancey M et al. Mammalian frataxin controls sulfur production and iron entry during de novo Fe_4_S_4_ cluster assembly. J Am Chem Soc 2013;135:733–40. 23265191 10.1021/ja308736e

[106] Gomes CM , Santos R. Neurodegeneration in Friedreich’s ataxia: from defective frataxin to oxidative stress. Oxid Med Cell Longev 2013;2013:487534. 23936609 10.1155/2013/487534 PMC3725840

[107] Cotticelli MG , Xia S, Lin D et al. Ferroptosis as a novel therapeutic target for Friedreich’s ataxia. J Pharmacol Exp Ther 2019;369:47–54. 30635474 10.1124/jpet.118.252759

[108] Tang D , Chen X, Kroemer G. Cuproptosis: a copper-triggered modality of mitochondrial cell death. Cell Res 2022;32:417–8. 35354936 10.1038/s41422-022-00653-7 PMC9061796

[109] Tsvetkov P , Coy S, Petrova B et al. Copper induces cell death by targeting lipoylated TCA cycle proteins. Science 2022;375:1254–61. 35298263 10.1126/science.abf0529 PMC9273333

[110] van der Bliek AM , Sedensky MM, Morgan PG. Cell biology of the mitochondrion. Genetics 2017;207:843–71. 29097398 10.1534/genetics.117.300262 PMC5676242

[111] Reznik E , Miller ML, Senbabaoglu Y et al. Mitochondrial DNA copy number variation across human cancers. Elife 2016;5:e10769. 26901439 10.7554/eLife.10769 PMC4775221

[112] Yang L , Lin X, Tang H et al. Mitochondrial DNA mutation exacerbates female reproductive aging via impairment of the NADH/NAD^+^ redox. Aging Cell 2020;19:e13206. 32744417 10.1111/acel.13206 PMC7511885

[113] Yang L , Long Q, Liu J et al. Mitochondrial fusion provides an ‘initial metabolic complementation’ controlled by mtDNA. Cell Mol Life Sci 2015;72:2585–98. 25708700 10.1007/s00018-015-1863-9 PMC11113443

[114] Yang L , Mei T, Lin X et al. Current approaches to reduce or eliminate mitochondrial DNA mutations. Sci China Life Sci 2016;59:532–5. 27106617 10.1007/s11427-014-0276-8

[115] El-Hattab AW , Scaglia F. Mitochondrial DNA depletion syndromes: review and updates of genetic basis, manifestations, and therapeutic options. Neurotherapeutics 2013;10:186–98. 23385875 10.1007/s13311-013-0177-6 PMC3625391

[116] Guo J , Duan L, He X et al. A combined model of human iPSC-derived liver organoids and hepatocytes reveals ferroptosis in DGUOK mutant mtDNA depletion syndrome. Adv Sci (Weinh) 2021;8:2004680. 34026460 10.1002/advs.202004680 PMC8132052

[117] Chen X , Zeh HJ, Kang R et al. Cell death in pancreatic cancer: from pathogenesis to therapy. Nat Rev Gastroenterol Hepatol 2021;18:804–23. 34331036 10.1038/s41575-021-00486-6

[118] Li C , Zhang Y, Liu J et al. Mitochondrial DNA stress triggers autophagy-dependent ferroptotic death. Autophagy 2021;17:948–60. 32186434 10.1080/15548627.2020.1739447 PMC8078708

[119] Dai E , Han L, Liu J et al. Ferroptotic damage promotes pancreatic tumorigenesis through a TMEM173/STING-dependent DNA sensor pathway. Nat Commun 2020;11:6339. 33311482 10.1038/s41467-020-20154-8 PMC7732843

[120] Zhang R , Kang R, Tang D. The STING1 network regulates autophagy and cell death. Signal Transduct Target Ther 2021;6:208. 34078874 10.1038/s41392-021-00613-4 PMC8172903

[121] Chan DC. Mitochondrial dynamics and its involvement in disease. Annu Rev Pathol 2020;15:235–59. 31585519 10.1146/annurev-pathmechdis-012419-032711

[122] Li C , Liu J, Hou W et al. STING1 promotes ferroptosis through MFN1/2-dependent mitochondrial fusion. Front Cell Dev Biol 2021;9:698679. 34195205 10.3389/fcell.2021.698679 PMC8236825

[123] Yu R , Lendahl U, Nister M et al. Regulation of mammalian mitochondrial dynamics: opportunities and challenges. Front Endocrinol (Lausanne) 2020;11:374. 32595603 10.3389/fendo.2020.00374 PMC7300174

[124] Zhang L , Wang Q, Liu W et al. The orphan nuclear receptor 4a1: a potential new therapeutic target for metabolic diseases. J Diabetes Res 2018;2018:9363461. 30013988 10.1155/2018/9363461 PMC6022324

[125] Liu M , Fan Y, Li D et al. Ferroptosis inducer erastin sensitizes NSCLC cells to celastrol through activation of the ROS-mitochondrial fission-mitophagy axis. Mol Oncol 2021;15:2084–105. 33675143 10.1002/1878-0261.12936 PMC8334255

[126] Zhou Y , Long Q, Wu H et al. Topology-dependent, bifurcated mitochondrial quality control under starvation. Autophagy 2020;16:562–74. 31234709 10.1080/15548627.2019.1634944 PMC6999642

[127] Liu J , Kuang F, Kroemer G et al. Autophagy-dependent ferroptosis: machinery and regulation. Cell Chem Biol 2020;27:420–35. 32160513 10.1016/j.chembiol.2020.02.005 PMC7166192

[128] Basit F , van Oppen LM, Schockel L et al. Mitochondrial complex I inhibition triggers a mitophagy-dependent ROS increase leading to necroptosis and ferroptosis in melanoma cells. Cell Death Dis 2017;8:e2716. 28358377 10.1038/cddis.2017.133 PMC5386536

[129] Chen X , Kang R, Kroemer G et al. Broadening horizons: the role of ferroptosis in cancer. Nat Rev Clin Oncol 2021;18:280–96. 33514910 10.1038/s41571-020-00462-0

[130] Alberio S , Mineri R, Tiranti V et al. Depletion of mtDNA: syndromes and genes. Mitochondrion 2007;7:6–12. 17280874 10.1016/j.mito.2006.11.010

[131] Li J , Cao F, Yin HL et al. Ferroptosis: past, present and future. Cell Death Dis 2020;11:88. 32015325 10.1038/s41419-020-2298-2 PMC6997353

[132] Fang X , Cai Z, Wang H et al. Loss of cardiac ferritin H facilitates cardiomyopathy via slc7a11-mediated ferroptosis. Circ Res 2020;127:486–501. 32349646 10.1161/CIRCRESAHA.120.316509

[133] Tang LJ , Zhou YJ, Xiong XM et al. Ubiquitin-specific protease 7 promotes ferroptosis via activation of the p53/TfR1 pathway in the rat hearts after ischemia/reperfusion. Free Radic Biol Med 2021;162:339–52. 33157209 10.1016/j.freeradbiomed.2020.10.307

[134] Song Y , Wang B, Zhu X et al. Human umbilical cord blood-derived MSCs exosome attenuate myocardial injury by inhibiting ferroptosis in acute myocardial infarction mice. Cell Biol Toxicol 2021;37:51–64. 32535745 10.1007/s10565-020-09530-8

[135] Park TJ , Park JH, Lee GS et al. Quantitative proteomic analyses reveal that GPX4 downregulation during myocardial infarction contributes to ferroptosis in cardiomyocytes. Cell Death Dis 2019;10:835. 31685805 10.1038/s41419-019-2061-8 PMC6828761

[136] Bai T , Li M, Liu Y et al. Inhibition of ferroptosis alleviates atherosclerosis through attenuating lipid peroxidation and endothelial dysfunction in mouse aortic endothelial cell. Free Radic Biol Med 2020;160:92–102. 32768568 10.1016/j.freeradbiomed.2020.07.026

[137] Wang H , An P, Xie E et al. Characterization of ferroptosis in murine models of hemochromatosis. Hepatology 2017;66:449–65. 28195347 10.1002/hep.29117 PMC5573904

[138] Martinet W , Coornaert I, Puylaert P et al. Macrophage death as a pharmacological target in atherosclerosis. Front Pharmacol 2019;10:306. 31019462 10.3389/fphar.2019.00306 PMC6458279

[139] NaveenKumar SK , SharathBabu BN, Hemshekhar M et al. The role of reactive oxygen species and ferroptosis in heme-mediated activation of human platelets. ACS Chem Biol 2018;13:1996–2002. 29869870 10.1021/acschembio.8b00458

[140] Liu J , Liu W, Liu Y et al. New thiazolidinones reduce iron overload in mouse models of hereditary hemochromatosis and beta-thalassemia. Haematologica 2019;104:1768–81. 30792208 10.3324/haematol.2018.209874 PMC6717595

[141] Matsushita M , Freigang S, Schneider C et al. T cell lipid peroxidation induces ferroptosis and prevents immunity to infection. J Exp Med 2015;212:555–68. 25824823 10.1084/jem.20140857 PMC4387287

[142] Weiland A , Wang Y, Wu W et al. Ferroptosis and its role in diverse brain diseases. Mol Neurobiol 2019;56:4880–93. 30406908 10.1007/s12035-018-1403-3 PMC6506411

[143] Li Q , Han X, Lan X et al. Inhibition of neuronal ferroptosis protects hemorrhagic brain. JCI Insight 2017;2:e90777. 28405617 10.1172/jci.insight.90777 PMC5374066

[144] Alim I , Caulfield JT, Chen Y et al. Selenium drives a transcriptional adaptive program to block ferroptosis and treat stroke. Cell 2019;177:1262–79.e25. 31056284 10.1016/j.cell.2019.03.032

[145] Shinde A , Paez JS, Libring S et al. Transglutaminase-2 facilitates extracellular vesicle-mediated establishment of the metastatic niche. Oncogenesis 2020;9:16. 32054828 10.1038/s41389-020-0204-5 PMC7018754

[146] Bao WD , Zhou XT, Zhou LT et al. Targeting miR-124/Ferroportin signaling ameliorated neuronal cell death through inhibiting apoptosis and ferroptosis in aged intracerebral hemorrhage murine model. Aging Cell 2020;19:e13235. 33068460 10.1111/acel.13235 PMC7681046

[147] Rui T , Wang H, Li Q et al. Deletion of ferritin H in neurons counteracts the protective effect of melatonin against traumatic brain injury-induced ferroptosis. J Pineal Res 2021;70:e12704. 33206394 10.1111/jpi.12704

[148] Zhou J , Jin Y, Lei Y et al. Ferroptosis is regulated by mitochondria in neurodegenerative diseases. Neurodegener Dis 2020;20:20–34. 32814328 10.1159/000510083

[149] Reichert CO , de Freitas FA, Sampaio-Silva J et al. Ferroptosis mechanisms involved in neurodegenerative diseases. Int J Mol Sci 2020;21:8765. 33233496 10.3390/ijms21228765 PMC7699575

[150] Bao WD , Pang P, Zhou XT et al. Loss of ferroportin induces memory impairment by promoting ferroptosis in Alzheimer’s disease. Cell Death Differ 2021;28:1548–62. 33398092 10.1038/s41418-020-00685-9 PMC8166828

[151] Wang M , Liu CY, Wang T et al. (+)-Clausenamide protects against drug-induced liver injury by inhibiting hepatocyte ferroptosis. Cell Death Dis 2020;11:781. 32951003 10.1038/s41419-020-02961-5 PMC7502081

[152] Yamada N , Karasawa T, Takahashi M. Role of ferroptosis in acetaminophen-induced hepatotoxicity. Arch Toxicol 2020;94:1769–70. 32180037 10.1007/s00204-020-02714-5

[153] Song H , Zhang S, Sun X et al. Distinct iron deposition profiles of liver zones in various models with iron homeostasis disorders. Adv Sci (Weinh) 2018;5:1800866. 30479929 10.1002/advs.201800866 PMC6247051

[154] Yang L , Wang H, Yang X et al. Auranofin mitigates systemic iron overload and induces ferroptosis via distinct mechanisms. Signal Transduct Target Ther 2020;5:138. 32732975 10.1038/s41392-020-00253-0 PMC7393508

[155] Yu Y , Jiang L, Wang H et al. Hepatic transferrin plays a role in systemic iron homeostasis and liver ferroptosis. Blood 2020;136:726–39. 32374849 10.1182/blood.2019002907 PMC7414596

[156] You Y , Liu C, Liu T et al. FNDC3B protects steatosis and ferroptosis via the AMPK pathway in alcoholic fatty liver disease. Free Radic Biol Med 2022;193:808–19. 36336231 10.1016/j.freeradbiomed.2022.10.322

[157] Li X , Wang TX, Huang X et al. Targeting ferroptosis alleviates methionine-choline deficient (MCD)-diet induced NASH by suppressing liver lipotoxicity. Liver Int 2020;40:1378–94. 32145145 10.1111/liv.14428

[158] Tsurusaki S , Tsuchiya Y, Koumura T et al. Hepatic ferroptosis plays an important role as the trigger for initiating inflammation in nonalcoholic steatohepatitis. Cell Death Dis 2019;10:449. 31209199 10.1038/s41419-019-1678-y PMC6579767

[159] Chen J , Li X, Ge C et al. The multifaceted role of ferroptosis in liver disease. Cell Death Differ 2022;29:467–80. 35075250 10.1038/s41418-022-00941-0 PMC8901678

[160] Wang Y , Quan F, Cao Q et al. Quercetin alleviates acute kidney injury by inhibiting ferroptosis. J Adv Res 2021;28:231–43. 33364059 10.1016/j.jare.2020.07.007 PMC7753233

[161] Guo L , Zhang T, Wang F et al. Targeted inhibition of Rev-erb-alpha/beta limits ferroptosis to ameliorate folic acid-induced acute kidney injury. Br J Pharmacol 2021;178:328–45. 33068011 10.1111/bph.15283

[162] Su L , Jiang X, Yang C et al. Pannexin 1 mediates ferroptosis that contributes to renal ischemia/reperfusion injury. J Biol Chem 2019;294:19395–404. 31694915 10.1074/jbc.RA119.010949 PMC6916502

[163] Deng F , Sharma I, Dai Y et al. Myo-inositol oxygenase expression profile modulates pathogenic ferroptosis in the renal proximal tubule. J Clin Invest 2019;129:5033–49. 31437128 10.1172/JCI129903 PMC6819105

[164] Zhang J , Bi J, Ren Y et al. Involvement of GPX4 in irisin’s protection against ischemia reperfusion-induced acute kidney injury. J Cell Physiol 2021;236:931–45. 32583428 10.1002/jcp.29903

[165] Li Y , Cao Y, Xiao J et al. Inhibitor of apoptosis-stimulating protein of p53 inhibits ferroptosis and alleviates intestinal ischemia/reperfusion-induced acute lung injury. Cell Death Differ 2020;27:2635–50. 32203170 10.1038/s41418-020-0528-x PMC7429834

[166] Wang M , Mao C, Ouyang L et al. Long noncoding RNA LINC00336 inhibits ferroptosis in lung cancer by functioning as a competing endogenous RNA. Cell Death Differ 2019;26:2329–43. 30787392 10.1038/s41418-019-0304-y PMC6889193

[167] Yoshida M , Minagawa S, Araya J et al. Involvement of cigarette smoke-induced epithelial cell ferroptosis in COPD pathogenesis. Nat Commun 2019;10:3145. 31316058 10.1038/s41467-019-10991-7 PMC6637122

[168] Wang Y , Chen D, Xie H et al. AUF1 protects against ferroptosis to alleviate sepsis-induced acute lung injury by regulating NRF2 and ATF3. Cell Mol Life Sci 2022;79:228. 35391558 10.1007/s00018-022-04248-8 PMC11072094

[169] Wei S , Qiu T, Yao X et al. Arsenic induces pancreatic dysfunction and ferroptosis via mitochondrial ROS-autophagy-lysosomal pathway. J Hazard Mater 2020;384:121390. 31735470 10.1016/j.jhazmat.2019.121390

[170] Li D , Jiang C, Mei G et al. Quercetin alleviates ferroptosis of pancreatic beta cells in type 2 diabetes. Nutrients 2020;12:2954. 32992479 10.3390/nu12102954 PMC7600916

[171] Kuang F , Liu J, Li C et al. Cathepsin B is a mediator of organelle-specific initiation of ferroptosis. Biochem Biophys Res Commun 2020;533:1464–9. 33268027 10.1016/j.bbrc.2020.10.035

[172] Liu K , Liu J, Zou B et al. Trypsin-mediated sensitization to ferroptosis increases the severity of pancreatitis in mice. Cell Mol Gastroenterol Hepatol 2022;13:483–500. 34562639 10.1016/j.jcmgh.2021.09.008 PMC8688567

[173] Wilmanski T , Zhou X, Zheng W et al. Inhibition of pyruvate carboxylase by 1alpha,25-dihydroxyvitamin D promotes oxidative stress in early breast cancer progression. Cancer Lett 2017;411:171–81. 29024812 10.1016/j.canlet.2017.09.045 PMC5763507

[174] Ye X , Weinberg RA. Epithelial-mesenchymal plasticity: a central regulator of cancer progression. Trends Cell Biol 2015;25:675–86. 26437589 10.1016/j.tcb.2015.07.012 PMC4628843

[175] Shinde A , Libring S, Alpsoy A et al. Autocrine fibronectin inhibits breast cancer metastasis. Mol Cancer Res 2018;16:1579–89. 29934326 10.1158/1541-7786.MCR-18-0151 PMC6511995

[176] Shinde A , Wilmanski T, Chen H et al. Pyruvate carboxylase supports the pulmonary tropism of metastatic breast cancer. Breast Cancer Res 2018;20:76. 30005601 10.1186/s13058-018-1008-9 PMC6045837

[177] Ma S , Dielschneider RF, Henson ES et al. Ferroptosis and autophagy induced cell death occur independently after siramesine and lapatinib treatment in breast cancer cells. PLoS One 2017;12:e0182921. 28827805 10.1371/journal.pone.0182921 PMC5565111

[178] Palmer LD , Jordan AT, Maloney KN et al. Zinc intoxication induces ferroptosis in A549 human lung cells. Metallomics 2019;11:982–93. 30968088 10.1039/c8mt00360b PMC6531343

[179] Probst L , Dachert J, Schenk B et al. Lipoxygenase inhibitors protect acute lymphoblastic leukemia cells from ferroptotic cell death. Biochem Pharmacol 2017;140:41–52. 28595877 10.1016/j.bcp.2017.06.112

[180] Montes de Oca Balderas P. Mitochondria-plasma membrane interactions and communication. J Biol Chem 2021;297:101164. 34481840 10.1016/j.jbc.2021.101164 PMC8503596

[181] Hu X , Duan T, Wu Z et al. Intercellular mitochondria transfer: a new perspective for the treatment of metabolic diseases. Acta Biochim Biophys Sin (Shanghai) 2021;53:958–60. 33890621 10.1093/abbs/gmab052

[182] Ng MYW , Wai T, Simonsen A. Quality control of the mitochondrion. Dev Cell 2021;56:881–905. 33662258 10.1016/j.devcel.2021.02.009

